# Combining LIANA and Tensor-cell2cell to decipher cell-cell communication across multiple samples

**DOI:** 10.1016/j.crmeth.2024.100758

**Published:** 2024-04-16

**Authors:** Hratch M. Baghdassarian, Daniel Dimitrov, Erick Armingol, Julio Saez-Rodriguez, Nathan E. Lewis

**Affiliations:** 1 Bioinformatics and Systems Biology Graduate Program, University of California, San Diego, La Jolla, CA 92093, USA; 2 Department of Pediatrics, University of California, San Diego, La Jolla, CA 92093, USA; 3 Heidelberg University, Faculty of Medicine, and Heidelberg University Hospital, Institute for Computational Biomedicine, 69120 Heidelberg, Germany; 4 Department of Bioengineering, University of California, San Diego, La Jolla, CA 92093, USA

**Keywords:** cell-cell communication, single-cell RNA sequencing, context dependent, tensor decomposition, ligand-receptor interactions, multiple conditions

## Abstract

In recent years, data-driven inference of cell-cell communication has helped reveal coordinated biological processes across cell types. Here, we integrate two tools, LIANA and Tensor-cell2cell, which, when combined, can deploy multiple existing methods and resources to enable the robust and flexible identification of cell-cell communication programs across multiple samples. In this work, we show how the integration of our tools facilitates the choice of method to infer cell-cell communication and subsequently perform an unsupervised deconvolution to obtain and summarize biological insights. We explain how to perform the analysis step by step in both Python and R and provide online tutorials with detailed instructions available at https://ccc-protocols.readthedocs.io/. This workflow typically takes ∼1.5 h to complete from installation to downstream visualizations on a graphics processing unit-enabled computer for a dataset of ∼63,000 cells, 10 cell types, and 12 samples.

## Highlights

• Integrated LIANA and Tensor-cell2cell for cell-cell communication analysis across samples
• Enables flexible selection of methods and resources for cell-cell communication inference
• Provides step-by-step analysis, accompanied by online tutorials in Python and R
• Demonstrates broad applicability to single-cell data in diverse biological conditions

## Motivation

Multiple cell-cell communication (CCC) tools exist, yet results are specific to the tool of choice due to the diverse assumptions made across computational frameworks. Moreover, tools are often limited to analyzing single samples or performing pairwise comparisons. As experimental design complexity and sample numbers continue to increase in single-cell datasets, so does the need for versatile methods to decipher cell-cell communication in such scenarios. By integrating LIANA and Tensor-cell2cell, we present a protocol that enables the use of a diverse array of tools and resources to assess interpretable CCC programs across multiple samples.

## Graphical abstract

**Figure:**
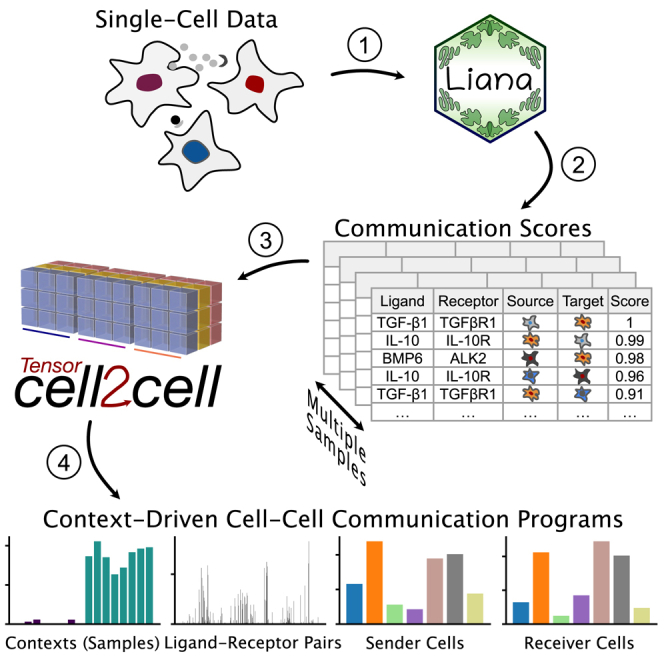

## Teaser

By integrating LIANA and Tensor-cell2cell, Baghdassarian et al. provide a unified protocol for unsupervised analysis of cell-cell communication (CCC) across multi-sample single-cell datasets. By facilitating CCC method selection and identification of context-driven communication programs, this approach garners insights into diverse biological processes. This work is accompanied by user-friendly tutorials for both Python and R.

## Introduction

Cell-cell communication (CCC) coordinates higher-order biological functions in multicellular organisms,^1^^,^^2^ dictating phenotypes in response to different contexts such as disease state, spatial location, and organismal life stage. In recent years, many tools have been developed to leverage single-cell and spatial transcriptomics data to study CCC events driving various biological processes.^2^^,^^3^^,^^4^ While each computational strategy contributes unique and valuable developments, many are tool specific and challenging to integrate due to the large number of different inference methods and resources housing prior knowledge.^2^^,^^5^^,^^6^^,^^7^ Moreover, most tools do not account for the relationships of coordinated CCC events (CCC programs) across different contexts,^8^ either disregarding context altogether by analyzing samples individually or being limited to pairwise comparisons. Thus, as the ability to generate large single-cell and spatial transcriptomics datasets and the interest in studying CCC programs continue to increase,^9^^,^^10^^,^^11^ the need to robustly decipher CCC is becoming essential.

### Comparison with other methods

A plethora of ligand-receptor (LR) methods have emerged, most of which were published with their own resources.^1^^,^^5^^,^^12^ Many of these provide distinct scoring functions to prioritize interactions, yet studies have reported low agreement between their predictions.^5^^,^^13^^,^^14^ Due to the lack of a gold standard, the benchmark of these methods remains limited,^2^^,^^5^ and it is challenging to choose the method that works best. To this end, in addition to providing multiple individual methods via ligand-receptor analysis framework (LIANA), we also enable their consensus, which we use in this protocol, under the assumption that the wisdom of the crowd is less biased than any individual method.^15^

While many methods exist to infer ligand-receptor interactions from a single sample, fewer approaches were designed to compare CCC interactions across conditions. These include CrossTalkeR,^16^ which utilizes network topological measures to compare communication patterns, CellPhoneDB,^17^ which accepts user-provided lists of differentially expressed genes to return relevant ligand-receptor interactions, and scDiffCom,^18^ which uses a combined permutation approach across both cell types and conditions. Still, the aforementioned approaches are limited to pairwise comparisons. Other approaches can directly compare CCC across more than two conditions; however, their analysis often relies on pairwise^19^ or targeted^20^ comparisons to integrate multiple samples. A key feature of Tensor-cell2cell is that it considers all samples simultaneously while preserving the relationships between ligand-receptor interactions and communicating cell-type pairs. Thus, Tensor-cell2cell preserves higher-order CCC relationships and translates those into mechanistic CCC programs of potentially interacting ligands, receptors, and communicating cell types.

### Development of the protocol

**Figure 1:**
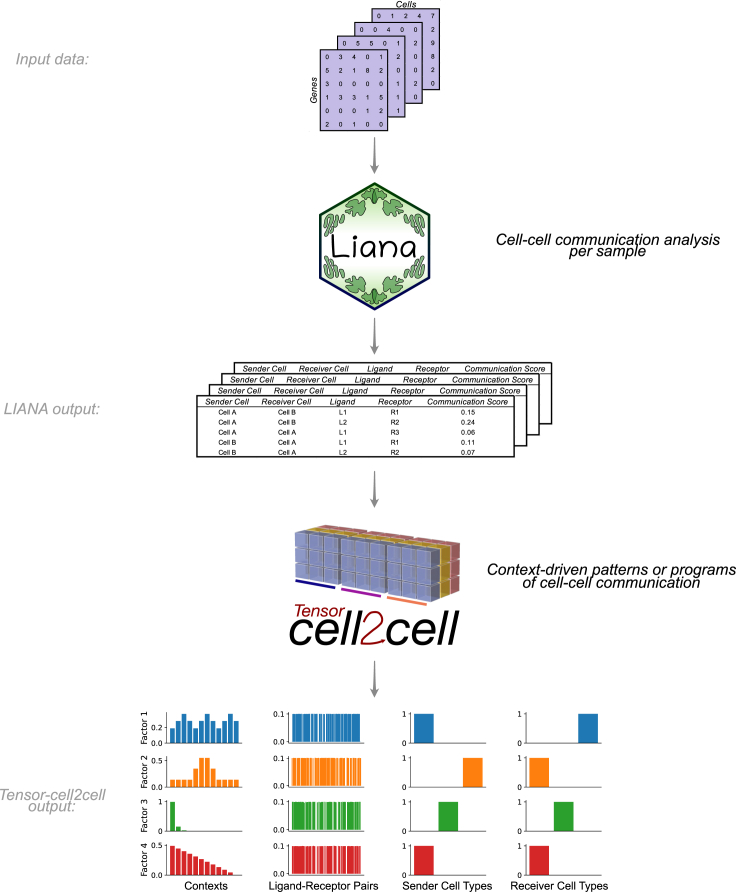
Integration of LIANA and Tensor-cell2cell to identify context-driven programs of cell-cell communication LIANA and Tensor-cell2cell can be used together to infer the molecular basis of cell-cell interactions by running analysis across multiple samples, conditions, or contexts. Given a method, resource, and expression data, LIANA outputs CCC scores for all interactions in a sample. We adapted both tools to be highly compatible with each other, so LIANA outputs can be directly passed to Tensor-cell2cell to detect the programs from the scores computed with LIANA. Tensor-cell2cell uses the communication scores generated for multiple samples to identify context-driven CCC programs.

We combine two independent yet highly complementary tools that leverage existing methods to enable robust and hypothesis-free analysis of context-driven CCC programs (Figure 1). LIANA^5^ is a computational framework that implements multiple available ligand-receptor resources (i.e., database of ligand-receptor interactions) and methods to analyze CCC. In particular, the user can employ LIANA to select any method and resource of choice or combine multiple approaches simultaneously to obtain consensus predictions. Tensor-cell2cell^12^ is a dimensionality reduction approach devised to uncover context-driven CCC programs across multiple samples simultaneously. Specifically, Tensor-cell2cell uses CCC scores inferred by any method and arranges the data into a four-dimensional (4D) tensor to capture the coordinated relationship between ligand-receptor interactions, communicating cell-type pairs, and samples. Together, LIANA and Tensor-cell2cell unify existing approaches to enable researchers to easily use their preferred CCC resource and method and subsequently analyze any number of samples into biologically relevant CCC insights without the additional complications of installing separate tools or reconciling discrepancies between them.

For this protocol, we adapted LIANA and Tensor-cell2cell to enable their smooth integration. Thus, our protocol demonstrates the concerted use of both tools, describes the insights they provide, and facilitates the interpretation of their outputs. We base this protocol on recent best practices for single-cell transcriptomics and CCC inference.^21^ We begin by processing the key inputs of our tools. Then, we guide the selection of methods and prior-knowledge resources to score intercellular communication using LIANA’s consensus method and resource to infer the potential CCC events for each sample. We use Tensor-cell2cell to summarize the intercellular communication events across samples, and we describe key technical considerations to enable consistent decomposition results. Finally, we guide the interpretation of the decomposition results and show multiple downstream analyses and visualizations to facilitate interpretation of the context-dependent CCC programs. For example, we illustrate how biologically relevant results can be obtained by coupling the outputs with pathway enrichment analyses. We also provide quick-start and in-depth online tutorials with detailed descriptions of all steps described in this protocol and their crucial parameters. All these materials are available in both Python and R at https://ccc-protocols.readthedocs.io/. While here we showcase an analysis on coronavirus disease 2019 (COVID-19) data, online tutorials also show applications on transcriptomics data of lupus peripheral blood mononuclear cells and spatial transcriptomics data of myocardial infarction, further demonstrating the adaptability of our combined tools. Collectively, these materials provide a comprehensive and flexible playbook to investigate CCC from single-cell transcriptomics.

### Applications of the protocol

LIANA and Tensor-cell2cell have been used for diverse purposes. LIANA was initially used to compare and evaluate different ligand-receptor methods in diverse biological contexts. Tensor-cell2cell was originally applied to link CCC programs with different severities of COVID-19 and autism spectrum disorder (ASD).^12^ Briefly, LIANA evaluated different methods and showed that they have limited agreement in terms of communication mechanisms,^5^^,^^12^ while Tensor-cell2cell revealed distinct CCC program dysregulations associated with severe COVID-19 specifically rather than moderate cases, as well as combinations of programs distinguishing ASD from neurotypical samples. Notably, LIANA provides a consensus resource and can aggregate multiple methods into consensus communication scores. Additionally, there is a natural complementarity between the two tools, as Tensor-cell2cell can use input scores from any CCC method (Figure 1) and generates consistent decomposition results across methods. Thus, our tools are highly generalizable and applicable to the analysis of any single-cell transcriptomics datasets. For example, LIANA has been used for the analysis of myocardial infarction^22^ and transforming growth factor β signaling in breast cancer,^23^ among others. Our tools are also applicable to other data modalities containing potentially interacting cell populations. Specifically, one can adapt LIANA or use existing spatial tools^24^ and combine their outputs with Tensor-cell2cell to generate spatially informed CCC insights across contexts. Similarly, one can also obtain metabolite-mediated intercellular interactions^25^^,^^26^ and decompose those into patterns across contexts with Tensor-cell2cell.^27^ One can also apply Tensor-cell2cell to extract CCC programs occurring at specific tissues^28^ or at a whole-body organism level.^28^^,^^29^ In this protocol, we focus on how one can leverage the different CCC methods and resources, generalized by LIANA, to infer context-dependent CCC programs with Tensor-cell2cell from single-cell transcriptomics data.

## Results

**Figure 2:**
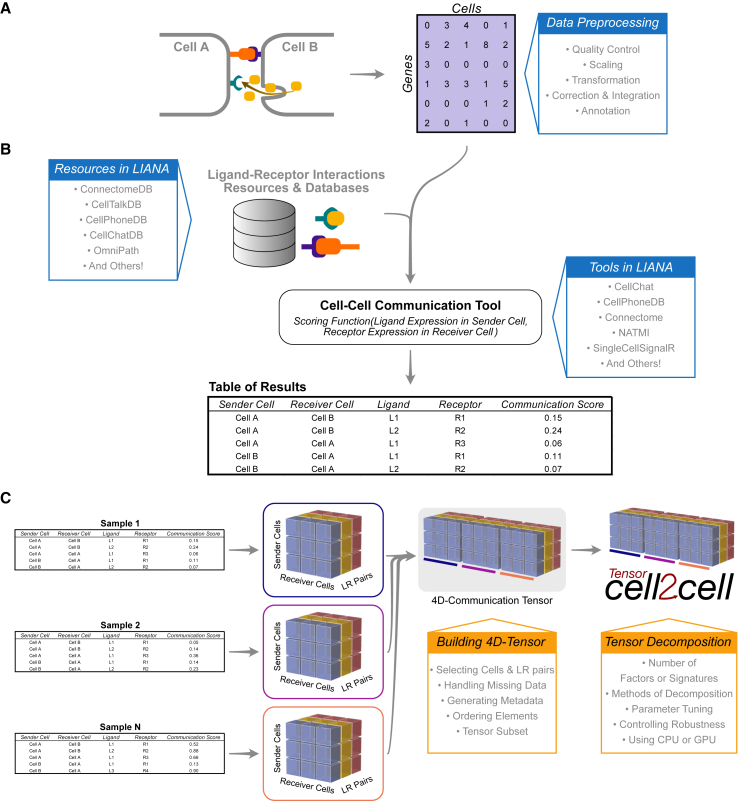
Overview of the protocol for inferring cell-cell communication through LIANA and Tensor-cell2cell Main inputs, steps, resources, and options are summarized for the distinct steps of this protocol. (A) A preprocessed gene expression matrix according to the best practices of single-cell analysis is expected as input (data preprocessing in the results section). (B) The input data are integrated with the ligand-receptor resources available in LIANA to infer cell-cell communication using any of the methods implemented in LIANA (inferring cell-cell communication in the results section). An output containing the cell-cell communication scores across all interactions per sample is generated. (C) The LIANA output is then directly passed to Tensor-cell2cell to build the respective communication tensor used by the tensor component analysis (building a 4D-communication tensor and running Tensor-cell2cell across samples in the results section). The output generated by Tensor-cell2cell can be later employed for other downstream analyses (downstream visualizations: making sense of the factors and pathway enrichment analysis: interpreting the context-driven communication in the results section).

In this section, we introduce our protocol (Figure 2) using Python. The same protocol is implemented in R and is available online at https://ccc-protocols.readthedocs.io/en/latest/notebooks/ccc_R/QuickStart.html.

### Step 1: Installation and environment setup

Install Anaconda or Miniconda through the official instructions at https://docs.anaconda.com/anaconda/install/index.html.

Then, open a terminal to create and activate a conda environment.

conda create -n ccc_protocols

conda activate ccc_protocols

If you will be using a graphics processing unit (GPU), then install PyTorch using conda.

conda install pytorch torchvision torchaudio pytorch-cuda=11.8 -c pytorch -c nvidia

Install Tensor-cell2cell, LIANA, and decoupler using PyPI.

pip install cell2cell liana decoupler

For fully reproducible runs of our tutorials in both Python and R, we have specified the required packages and their versions in the software requirements table (STAR Methods). You can also follow instructions in the environment setup section to install a clean virtual environment with all package requirements.

Notebooks to run this tutorial can be created by starting a Jupyter Notebook.

jupyter notebook

### Step 2: Initial setups

First, if you are using an NVIDIA GPU with Compute Unified Device Architecture (CUDA) cores, then set “use_gpu = True” and enable PyTorch with the following code block. Otherwise, set “use_gpu = False” or skip this part.

use_gpu = True

if use_gpu:

import tensorly as tl

 tl.set_backend('pytorch')

Then, import all the packages we will use in this tutorial.

import cell2cell as c2c

import liana as li

import pandas as pd

import decoupler as dc

import scanpy as sc

import matplotlib.pyplot as plt

%matplotlib inline

import plotnine as p9

import seaborn as sns

Afterward, specify the data and output directories.

data_folder = '../../data/quickstart/'

output_folder = '../../data/quickstart/outputs/'

c2c.io.directories.create_directory(data_folder)

c2c.io.directories.create_directory(output_folder)

We begin by loading the single-cell transcriptomics data. For this tutorial, we will use a lung dataset of 63,000 immune and epithelial cells across three control, three moderate, and six severe COVID-19 patients (Zenodo Data: https://doi.org/10.5281/zenodo.7706962).^30^ We use a convenient function to download the data and store it in the AnnData format, on which the scanpy^31^ package is built.

adata = c2c.datasets.balf_covid(data_folder + '/Liao-BALF-COVID-19.h5ad')

### Step 3: Data preprocessing

Data preprocessing is crucial for the correct application of this (Figure 2A). Here, we only highlight the essential steps. However, other aspects of data preprocessing should be considered and performed according to the best practices of single-cell analysis (https://github.com/theislab/single-cell-best-practices).

#### Quality control (timing: <5 min)

The loaded data have already been preprocessed to a degree and come with cell annotations. Nevertheless, we highlight some of the key steps. To mitigate noise, we filter non-informative cells and genes.

sc.pp.filter_cells(adata, min_genes=200)

sc.pp.filter_genes(adata, min_cells=3)

We additionally remove a high mitochondrial content.

adata.var['mt'] = adata.var_names.str.startswith('MT-')

sc.pp.calculate_qc_metrics(adata,

qc_vars=['mt'],

percent_top=None,

log1p=False,

inplace=True)

adata = adata[adata.obs.pct_counts_mt < 15, :]

This is followed by removing cells with a high number of total unique molecular identifier (UMI) counts, potentially representing more than one single cell (doublets):

adata = adata[adata.obs.n_genes < 5500,:]

Caution: Here, we covered the absolute basics. We omit other common practice steps, such as the removal of doublets and cells with high ribosomal content and the correction of ambient RNA. Additionally, in certain scenarios, particularly in those where technical variation is expected to be notable, the application of quality control steps by sample is desirable.^21^

#### Normalization (timing: <2 min)

We have now removed the majority of noisy readouts and can proceed to count normalization, as most CCC tools typically use normalized count matrices as input. Normalized counts are usually obtained in two essential steps, the first being count depth scaling, which ensures that the measured count depths are comparable across cells. This is then usually followed up with log1p transformation, which stabilizes the variance of the counts and enables the use of linear metrics downstream.

# Save the raw counts to a layer

adata.layers["counts"] = adata.X.copy()

# Normalize the data

sc.pp.normalize_total(adata, target_sum=1e4)

sc.pp.log1p(adata)

Critical: A key parameter of this command is as follows:

• “target_sum” ensures that after normalization, each observation (cell) has a total count equal to that number.

These normalization steps ensure that the aggregation of cells into cell types, a common practice for CCC inference, is done on comparable cells with approximately normally distributed feature values.

Troubleshooting: Expression matrices with “not a number” (nan), negative, or infinity (inf) values cause errors. Users should stick to common normalization techniques, and any nan, negative, or inf values must be filled to avoid errors.

### Step 4: Inferring CCC

**Figure 3:**
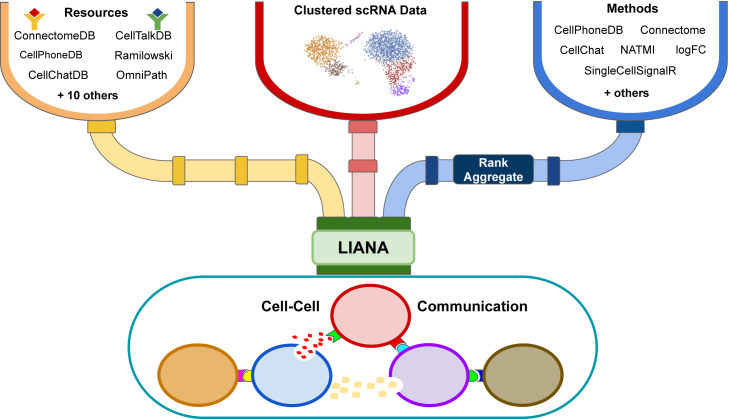
LIANA is a user-friendly and modular ligand-receptor analysis framework LIANA provides a variety of methods and resources to infer cell-cell communication, making it easy to use multiple existing methods in a coherent manner. It also provides consensus scores and resources to provide generalized results. Figure was adapted from Dimitrov et al.^5^

Following preprocessing of the single-cell transcriptomics data, we proceed to the inference of potential CCC events (Figure 2B). In this case, we will use LIANA to infer the ligand-receptor interactions for each sample. LIANA is available in Python and R and supports Scanpy, SingleCellExperiment, and Seurat objects as input. LIANA is highly modularized, and it natively implements the formulations of several methods, including CellPhoneDBv2,^32^ Connectome,^33^ log2 fold change (log2FC), NATMI,^34^ SingleCellSignalR,^35^ CellChat,^19^ and a geometric mean, as well as a consensus score in the form of a rank aggregate^36^ from any combination of methods (Figure 3). The high modularity of LIANA further enables the straightforward addition of any other ligand-receptor method.

LIANA classifies the scoring functions from the different methods into two categories: those that infer the “magnitude” and “specificity” of interactions. The magnitude of an interaction is a measure of the strength of the interaction, and the specificity of an interaction is a measure of how specific an interaction is to a given pair of cell groups. Generally, these categories are complementary, and the magnitude of the interaction is often in agreement with the specificity of the interaction. In other words, a ligand-receptor interaction with a high magnitude score in a given pair of cell types is likely to also be specific, and vice versa.

#### Selecting a method to infer CCC

While there are many commonalities between the different methods implemented in LIANA, there also are many variations and different assumptions affecting how the magnitude and specificity scores are calculated (see STAR Methods). These variations can result in limited agreement in inferred predictions when using different CCC methods.^5^^,^^13^^,^^14^ To this end, in LIANA, we additionally provide a “rank_aggregate” score, which can be used to aggregate any of the scoring functions above into a consensus score.

By default, LIANA calculates an aggregate rank using a re-implementation of the RobustRankAggregate method^36^ and generates a probability distribution for ligand-receptors that are ranked consistently better than expected under a null hypothesis (see STAR Methods). The consensus of ligand-receptor interactions across methods can therefore be treated as a *p* value. We show in detail how LIANA’s rank aggregate or any of the individual methods can be used to infer communication events from a single sample or context at “Python Tutorial 02 Infer-Communication-Scores” (https://ccc-protocols.readthedocs.io/en/latest/notebooks/ccc_python/02-Infer-Communication-Scores.html).

Critical: When using LIANA with Tensor-cell2cell, we recommend selecting a scoring function that reflects the magnitude of the interactions, as how the interactions’ specificity relates to changes across samples is unclear. In this protocol, we will use the “magnitude_rank” scoring function from LIANA, under the assumption that ensemble approaches are potentially less biased than any single method alone.^15^

We further show that Tensor captures consistent CCC programs when using different methods and add a tutorial to explore method consistency on any dataset: https://ccc-protocols.readthedocs.io/en/latest/notebooks/ccc_python/S3B_Score_Consistency.html.

Troubleshooting: The default decomposition method of Tensor-cell2cell is a non-negative tensor component analysis, which, as implied, expects non-negative values as the inputs. Thus, when selecting the method of choice, make sure that you do not have negative CCC scores. If so, you can replace them by zeros or the minimum positive value.

#### Selecting ligand-receptor resources

When considering ligand-receptor prior-knowledge resources, a common theme is the trade-off between coverage and quality, and similarly, each resource comes with its own biases.^5^ LIANA takes advantage of OmniPath,^37^ which includes expert-curated resources of CellPhoneDBv2,^32^ CellChat,^19^ ICELLNET,^38^ connectomeDB2020,^34^ and CellTalkDB,^39^ as well as 10 others.^5^^,^^37^ LIANA further provides a consensus expert-curated resource from the aforementioned five resources, along with some curated interactions from SignaLink.^40^ In this protocol, we will use the consensus resource from LIANA, though any of the other resources are available via LIANA, and one can also use LIANA with their own custom resource.

Selecting any of the lists of ligand-receptor pairs in LIANA can be done through the following command.

lr_pairs = li.resource.select_resource('consensus')

Here, “consensus” indicates the use of LIANA’s consensus resource, but it can be replaced by any other available resource (e.g., “cellphonedb,” “cellchatdb,” “connectomeDB,” etc.).

Note that any of the resources available in LIANA can be used by passing them as a string to “resource_name.” All of LIANA’s resources can be listed with “li.resource.show_resources().” Users can also provide custom resources as a pandas DataFrame to run in LIANA so long as they are formatted the same as other resources (i.e., include two columns named ligand and receptor, containing the respective partners in the ligand-receptor interactions). Hence, users may pass a dataframe containing a personalized list of interactions to liana using the “resource” parameter in the next “rank_aggregate” function below.

Troubleshooting: Users should choose a resource with gene identifiers and an organism that corresponds to that of their data. By default, LIANA uses human gene symbol identifiers but additionally provides a murine resource as well as functionalities to convert via orthology to other organisms.

#### Running LIANA for each sample (timing: 4 min)

Here, we will run LIANA’s “rank_aggregate” with six methods (by default, CellPhoneDBv2, CellChat, SingleCellSignalR, NATMI, Connectome, and log2FC) on all of the samples in the dataset.

li.mt.rank_aggregate.by_sample(adata,

sample_key='sample_new',

groupby='celltype', resource_name='consensus',

expr_prop=0.1,

min_cells=5,

n_perms=100,

use_raw=False,

verbose=True,

inplace=True

)

Critical: Key parameters here are as follows:

• “adata” stands for AnnData, the data format used by scanpy.^31^
• “sample_key” corresponds to the sample identifiers, available as a column in the “adata.obs” dataframe.
• “groupby” corresponds to the cell group label stored in “adata.obs.”
• “resource_name” is the name of any of the resources available via LIANA.
• “expr_prop” is the expression proportion threshold (in terms of cells per cell type expressing the protein) for any protein subunit involved in the interaction, according to which we keep or discard the interactions.
• “min_cells” is the minimum number of cells per cell type required for a cell type to be considered in the analysis.
• “n_perms” is the number of permutations for *p* value estimation.
• “use_raw” is a Boolean that indicates whether to use the “adata.raw” slot; here, the log-normalized counts are assigned to “adata.X,” and other options include passing the name of a layer via the “layer” parameter or using the counts stored in “adata.raw.”

Critical: LIANA considers interactions as occurring only if the ligand and receptor, and all of their subunits, are expressed in at least 10% of the cells (by default) in both clusters involved in the interaction. Any interactions that do not pass these criteria are not returned by default. To return those, the user can use the “return_all_lrs” parameter. These results will later be used to generate a tensor of ligand-receptor interactions across contexts that will be decomposed into CCC programs by Tensor-Cell2cell. Thus, how non-expressed interactions are handled is critical to consider when building the tensor later on (see “Python Tutorial 03 Generate-Tensor” (https://ccc-protocols.readthedocs.io/en/latest/notebooks/ccc_python/03-Generate-Tensor.html).

One can visualize the output as a dot plot while including every sample in the dataset.

li.pl.dotplot_by_sample(adata=adata,

colour='magnitude_rank',

size='specificity_rank',

source_labels=["B", "pDC", "Epithelial"],

target_labels=["Macrophages", "Mast", "pDC", "NK"],

ligand_complex=['VIM', 'SCGB3A1'],

receptor_complex=['CD44', 'MARCO'],

sample_key='sample_new',

inverse_colour=True,

inverse_size=True,

figure_size=(14, 10),

size_range=(1, 6),

)

Key parameters here are as follows:

• “source_labels” is a list containing the names of the sender cells of interest.
• “target_labels” is a list containing the names of the receiver cells of interest.
• “ligand_complex” is a list containing the names of the ligands of interest.
• “receptor_complex” is a list containing the names of the receptors of interest.
• “sample_key” is a string containing the column name where samples are specified.

**Figure 4:**
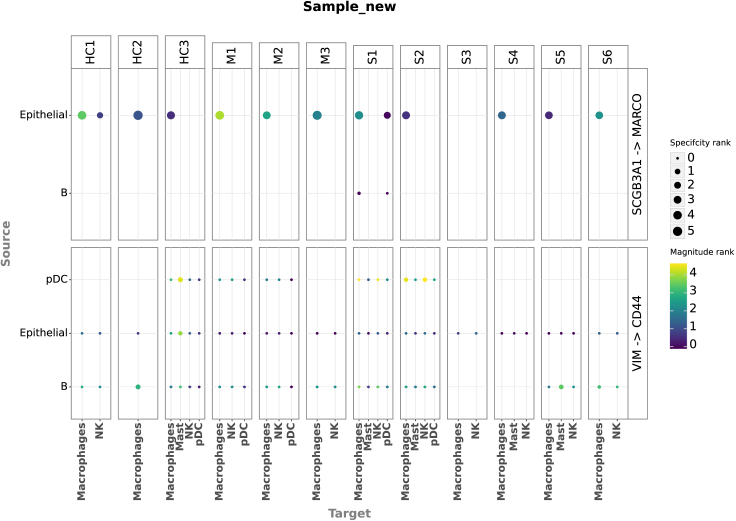
Dot plot of cell-cell communication between immune cells per sample Here, sender and receiver cells are represented as source and target (y and x axes, respectively). Each major column groups cells by sample, while each major row groups cells by the ligand-receptor interaction they are using. Dot size represents the specificity (ranks) assigned by LIANA, while the color represents the magnitude (ranks) of the interaction.

This command leads to the generation of Figure 4.

Pause point: We can export the LIANA results by sample to a CSV and save them for later use.

adata.uns['liana_res'].to_csv(output_folder + '/LIANA_by_sample.csv', index=False)

Alternatively, one could just export the whole AnnData object, together with the ligand-receptor results stored at “adata.uns[‘liana_res’].”

adata.write_h5ad(output_folder + '/adata_processed.h5ad', compression='gzip')

### Step 5: Comparing CCC across multiple samples

#### Building a 4D-communication tensor (timing: <1 min)

First, we generate a list containing all samples from our AnnData object. For visualization purposes, we sort them according to COVID-19 severity. Here, we can find the names of each of the samples in the “sample_new” column of the adata.obs information.

sorted_samples = sorted(adata.obs['sample_new'].unique())

Tensor-cell2cell performs a tensor decomposition to find context-dependent patterns of CCC. It builds a 4D-communication tensor, which, in this case, is built from the communication scores obtained from LIANA for every combination of ligand-receptor and sender-receiver cell pairs per sample (Figures 2B and 2C). For this protocol and associated tutorials, we implemented a function that facilitates building this communication tensor.

tensor = li.multi.to_tensor_c2c(liana_res=adata.uns['liana_res'],

sample_key='sample_new',

source_key='source',

target_key='target',

ligand_key='ligand_complex',

receptor_key='receptor_complex',

score_key='magnitude_rank',

inverse_fun=lambda x: 1 - x,

how='outer',

outer_fraction=1/3.,

context_order=sorted_samples,

)

Troubleshooting: Since the “magnitude_rank” from LIANA represents a score where the values closest to 0 represent the most probable communication events, we need to invert the communication scores to use it with Tensor-cell2cell. See the parameter “inverse_fun” below for further details for transforming this score.

Critical: Key parameters here are as follows:

• “liana_res” is the dataframe containing the results from LIANA, usually located in “adata.uns[‘liana_res’].” We can pass directly the AnnData object to the parameter adata to this function. If the AnnData object is passed, then we do not need to specify the liana_res parameter.
• “sample_key,” “source_key,” “target_key,” “ligand_key,” “receptor_key,” and “score_key” are the column names in the dataframe containing the samples, sender cells, receiver cells, ligands, receptors, and communication scores, respectively. Each row of the dataframe contains a unique combination of these elements.
• “inverse_fun” is the function we use to convert the communication score before building the tensor. In this case, the “magnitude_rank” score generated by LIANA considers low values as the most important ones, ranging from 0 to 1. In contrast, Tensor-cell2cell requires higher values to be the most important scores, so here we pass a function (lambda x: 1 − x) to adapt LIANA’s magnitude-rank scores (subtracts LIANA’s score from 1). If “None” is passed instead, then no transformation will be performed on the communication score. If using other scores coming from one of the methods implemented in LIANA, then a similar transformation can be done depending on the parameters and assumptions of the scoring method.
• “how” controls “which” ligand-receptor pairs and cell types to include when building the tensor. This decision depends on whether the missing values across a number of samples for both ligand-receptor interactions and sender-receiver cell pairs are considered to be biologically relevant. Options are as follows:
  o “inner” is the most strict option since it only considers cell types and ligand-receptor pairs that are present in all contexts (intersection).
  o “outer” considers all cell types and ligand-receptor pairs that are present across contexts (union).
  o “outer_lrs” considers only cell types that are present in all contexts (intersection) but all ligand-receptor pairs that are present across contexts (union).
  o “outer_cells” considers only ligand-receptor pairs that are present in all contexts (intersection) but all cell types that are present across contexts (union).
• “outer_fraction” controls the elements to include in the union scenario of the how options. Only elements that are present at least in this fraction of samples/contexts will be included. When this value is 0, the tensor includes all elements across the samples. When this value is 1, it acts as using how = “inner.”
• “context_order” is a list specifying the order of the samples. The order of samples does not affect the results, but it is useful for posterior visualizations.

We can check the shape of this tensor to verify the number of samples, ligand-receptor pairs, sender cells, and receiver cells, respectively:

tensor.shape

In addition, optionally, we can generate the metadata for coloring the elements in each of the tensor dimensions (i.e., for each of the contexts/samples, ligand-receptor pairs, sender cells, and receiver cells) in posterior visualizations. These metadata correspond to dictionaries for each of the dimensions containing the elements and their respective major groups, such as a signaling categories for a ligand-receptor interactions, a hierarchically more granular cell type, or a disease condition for a sample. In cases where we do not account for such information, we do not need to generate such dictionaries.

For example, we can build a dictionary for the contexts/samples dictionary by using the metadata in the AnnData object. In this example dataset, we can find samples in the column “sample_new,” while their major groups (representing COVID-19 severity) are found in the column “condition.”

context_dict = adata.obs.sort_values(by='sample_new') \

.set_index('sample_new')['condition'] \

.to_dict()

Then, the metadata can be generated with:

dimension_dicts = [context_dict, None, None, None] meta_tensor = c2c.tensor.generate_tensor_metadata(interaction_tensor=tensor, metadata_dicts=dimension_dicts, fill_with_order_elements=True)

Notice that the “None” elements in the variable dimensions_dicts represent the dimensions where we are not including additional metadata. If you want to include metadata about major groups for those dimensions, then you have to replace the corresponding “None” with a dictionary as described before.

Pause point: We can export our tensor and its metadata for performing the tensor decomposition later:

c2c.io.export_variable_with_pickle(variable=tensor,

filename=output_folder + '/Tensor.pkl')

c2c.io.export_variable_with_pickle(variable=meta_tensor,

filename=output_folder + '/Tensor-Metadata.pkl')

tensor = c2c.io.read_data.load_tensor(output_folder + '/Tensor.pkl')

meta_tensor = c2c.io.load_variable_with_pickle(output_folder + '/Tensor-Metadata.pkl')

Then, we can load them with:

tensor = c2c.io.read_data.load_tensor(output_folder + '/Tensor.pkl')

meta_tensor = c2c.io.load_variable_with_pickle(output_folder + '/Tensor-Metadata.pkl')

#### Running Tensor-cell2cell across samples (timing: 5 min with a “regular” run or 40 min with a “robust” run, using a GPU in both cases)

Now that we have built the tensor and its metadata, we can run tensor component analysis via Tensor-cell2cell with one simple command that we implemented for our unified tools.

c2c.analysis.run_tensor_cell2cell_pipeline(interaction_tensor=tensor,

tensor_metadata=meta_tensor,

rank=None,

tf_optimization='robust',

random_state=0,

device='cuda',

output_folder=output_folder,

)

**Figure 5:**
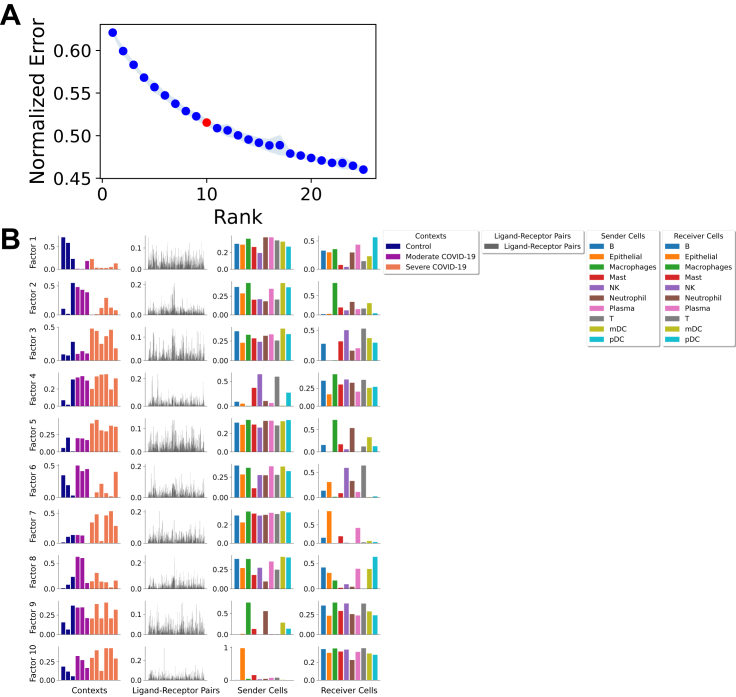
Cell-cell communication programs obtained by combining LIANA and Tensor-cell2cell (A) Elbow analysis to select an optimal number of factors (rank of the tensor). The red dot represents the number selected by Tensor-cell2cell. (B) After inferring cell-cell communication with LIANA from the COVID-19 data and running a tensor component analysis with Tensor-cell2cell, 10 factors were obtained (rows here), each of which represents a different cell-cell communication program. Within a factor, loadings (y axis) are reported for each element (x axis) in every tensor dimension (columns). Elements here are colored by their major groups as indicated in the legend.
• “tf_optimization” indicates whether running the analysis in the regular or the robust way. It essentially controls the convergence parameters of the tensor decomposition. The former employs less strict convergence parameters to obtain optimal results than the latter, which is also translated into a faster generation of results.
• “random_state” is the seed for randomization. It controls the randomization used when initializing the optimization algorithm that performs the tensor decomposition. It is useful for reproducing the same result every time that the analysis is run. If “None,” then a different randomization will be used each time.
• “device” indicates whether we are using the “cpu” or a GPU with “cuda” cores. See the installation section of this tutorial for instructions to enable the use of GPU(s).

Critical: Key parameters of this command are as follows:

• “rank” is the number of factors or latent patterns we want to obtain from the analysis. You can either indicate a specific number or leave it as “None” to perform the decomposition with a suggested number from an elbow analysis (Figure 5A).

This command will output three main results: a figure with the elbow analysis for suggesting a number of factors for the decomposition (only if “rank = None”), a figure with the loadings assigned to each element within a tensor dimension per factor obtained, and an Excel file containing the values of these loadings. Figure 5A represents the elbow plot generated in this case, while Figure 5B shows the loadings assigned to each tensor dimension per factor.

Troubleshooting: Elbow analysis returns a rank equal to one, or the curve is increasing instead of decreasing. This may be due to high sparsity in the tensor. The sparsity can be decreased by re-building the 4D tensor after re-running LIANA (running LIANA for each sample) with a smaller “expr_prop” (e.g., “expr_prop = 0.05”) or by only re-building the tensor (building a 4D-communication tensor) with a higher “outer_fraction” (e.g., “outer_fraction = 0.8”).

#### Downstream visualizations: Making sense of the factors (timing: <2 min)

The figure representing the loadings in each factor generated in the previous section can be interpreted by interconnecting all dimensions within a single factor (Figure 5B). For each factor, we obtain four vectors that represent the sample, ligand-receptor interaction, sender cell type, and receiver cell-type loadings. These loadings are the relative importance of each element in each dimension of the original tensor. All these vectors together define a CCC program in which high loadings represent key cells and mediators. By focusing on sample loadings associated with a given condition label, we can thus identify the cell types and interactions also associated with that label. Relevant interactors can be interpreted according to their loadings (i.e., ligand-receptor pairs, sender cells, and receiver cells with high loadings play a more prominent role in an identified CCC program). Ligands in high-loading ligand-receptor pairs are sent predominantly by high-loading sender cells and interact with the cognate receptors on the high-loadings receiver cells. In this regard, the program would be predominantly driven by changes in the receptor expression of receiver cells such as macrophages, neutrophils, and myeloid dendritic cells.

We can access the loading values of samples in each of the factors with

tensor.factors['Contexts']

In this case, we obtain a dataframe where rows represent the samples, columns the factors generated by the decomposition, and entries the loadings of each element within the corresponding factor. We can also access the loadings for the elements in the other dimensions by replacing “Contexts” with “Ligand-Receptor Pairs,” “Sender Cells,” or “Receiver Cells.” Then, we can use these loadings to perform various downstream analyses.

**Figure 6:**
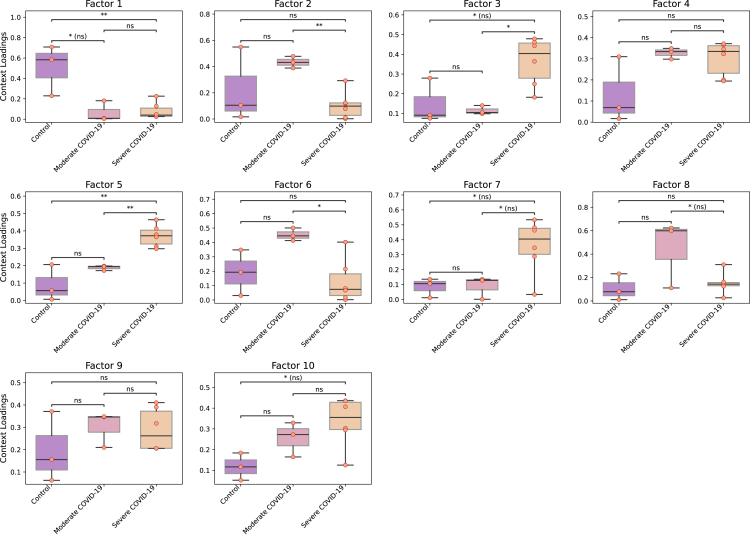
Identifying patterns and differences across groups of conditions Context or sample loadings can be used to compare statistically different condition groups within the same cell-cell communication program. Here, COVID-19 patients are grouped by severity, and pairwise t tests are performed. Here, ∗ and ∗∗ indicate *p* values lower than 0.05 and 0.01, respectively, while ns means not-significant (or *p* value greater than 0.05). The case of “ns” indicates that the significance is lost after multiple test correction (false discovery rate, in this case).

For example, we can use loadings to compare groups of samples (Figure 6) with boxplots and statistical tests.

groups_order = ['Control', 'Moderate COVID-19', 'Severe COVID-19']

fig_filename = output_folder + '/BALF-Severity-Boxplots.pdf'

_ = c2c.plotting.context_boxplot(context_loadings=tensor.factors['Contexts'],

metadict=context_dict,

nrows=3,

figsize=(16, 12),

group_order=groups_order,

statistical_test='t-test_ind',

pval_correction='fdr_bh',

cmap='plasma',

verbose=False,

filename=fig_filename

)

Critical: In this case, we can change the statistical test and the multiple-test correction with the parameters “statistical_test” and “pval_correction.” Here, we used an independent t test and a Benjamini-Hochberg correction. Additionally, we can set “verbose = True” to print exact test statistics and *p* values.

We can also generate heatmaps for the elements with loadings above a certain threshold in a given dimension (Figure S1). Furthermore, we can cluster these elements by the similarity of their loadings across all factors.

fig_filename = output_folder + '/Clustermap-LRs.pdf'

_ = c2c.plotting.loading_clustermap(loadings=tensor.factors['Ligand-Receptor

Pairs'],

loading_threshold=0.1,

use_zscore=False,

figsize=(28, 8),

filename=fig_filename,

row_cluster=False

)

Troubleshooting: Note that here, we plot the loadings of the dimension representing the ligand-receptor pairs. In addition, we prioritize the pairs with high loadings using the parameter “loading_threshold = 0.1.” In this case, the elements are included only if they are greater than or equal to that threshold in at least one of the factors. If we use “loading_threshold = 0,” then we would consider all of the elements. Considering all of the elements would require modifying the parameter “figsize” to enlarge the figure.

Caution: Changing the parameter “use_zscore” to “True” would standardize the loadings of one element across all factors. This is useful to compare an element across factors and highlight the factors in which that element is most important. Modifying “row_cluster” to “True” would also cluster the factors depending on the elements that are important in each of them.

Furthermore, factor-specific networks of cell-cell interactions (Figure S2) can be visualized by using the loadings of sender and receiver cells.

threshold = 0.075

c2c.plotting.ccc_networks_plot(tensor.factors,

included_factors=['Factor 3', 'Factor 5', 'Factor 10'],

ccc_threshold=threshold, # Only important communication

nrows=1,

panel_size=(16, 16), # This changes the size of each figure panel.

filename=output_folder + 'Factor-Networks.pdf',

)

Critical: Key parameters of this command are as follows:

• “included_factors” is a list of factors to plot. If “None” is passed, then all factor-specific networks are shown.
• “ccc_threshold” is a loading value to set as threshold to select key cell-cell interactions. This threshold filters the outer products between sender and receiver cells, and it can be either arbitrary or determined as shown in the online tutorials.

### Step 6: Pathway enrichment analysis: Interpreting the context-driven communication

The decomposition of ligand-receptor interactions across samples into loadings associated with the conditions reduces the dimensionality of the inferred interactions substantially. Nevertheless, we are still working with 1,054 interactions across multiple factors associated with the disease labels. To this end, as is commonly done when working with omics data types, we can perform pathway enrichment analysis to identify the general biological processes of interest. By using the loadings for each ligand-receptor pair (Figure 5B), we can rank them within each factor and use this ranking as input to enrichment analysis. Pathway enrichment thus serves two purposes: it further reduces the dimensionality of the inferred interactions and it enhances the biological interpretability of the inferred interactions.

Here, we show the application of classical gene set enrichment analysis (GSEA) on the ligand-receptor loadings. We use GSEA^41^ with KEGG Pathways,^42^ as well as a multivariate linear regression from decoupler-py^43^ with the PROGENy pathway resource.^44^

First, we assign ligand-receptor loadings to a variable.

lr_loadings = tensor.factors['Ligand-Receptor Pairs']

#### Classic pathway enrichment (timing: <1 min)

For the pathway enrichment analysis, we use ligand-receptor pairs instead of individual genes. KEGG was initially designed to work with sets of genes, so first we need to generate ligand-receptor sets for each of its pathways. A ligand-receptor pair is assigned as part of a pathway set if all of the genes in the pair are part of the gene set of such a pathway.

Note that we use the “lr_pairs” database that we loaded in the selecting ligand-receptor resources section.

# Generate list with ligand-receptors pairs in DB

lr_list = ['^'.join(row) for idx, row in lr_pairs.iterrows()]

# Specify the organism and pathway database to use for building the LR set

organism = "human"

pathwaydb = "KEGG"

# Generate ligand-receptor gene sets

lr_set = c2c.external.generate_lr_geneset(lr_list,

complex_sep='_',

lr_sep='^',

organism=organism,

pathwaydb=pathwaydb,

readable_name=True,

output_folder=output_folder

)

Critical: Key parameters of this command are as follows:

• “complex_sep” indicates the symbol separating the gene names in the protein complex.
• “lr_sep” is the symbol separating a ligand and a receptor complex.
• “organism” is the organism matching the gene names in the single-cell dataset. It could be either “human” or “mouse.”
• “pathwaydb” is the name of the database to be loaded, provided with the cell2cell package. Options are “GOBP,” “KEGG,” and “Reactome.”

Run GSEA via cell2cell, which calls the “gseapy.prerank” function internally.

pvals, scores, gsea_df = c2c.external.run_gsea(loadings=lr_loadings,

lr_set=lr_set,

output_folder=output_folder,

weight=1,

min_size=15,

permutations=999,

processes=6,

random_state=6,

significance_threshold=0.05,

)

Critical: Key parameters of this command are as follows:

• “lr_set” is a dictionary associating pathways (keys) with sets of ligand-receptor pairs (values).
• “weight” represents the original parameter p in GSEA. It is an exponent that controls the importance of the ranking values (loadings, in our case).
• “min_size” indicates the minimum number of LR pairs that a set has to contain to be considered in the analysis.
• “permutations” indicates the number of permutations to perform to generate the null distribution.
• “random_state” is the reproducibility seed.
• “significance_threshold” is the *p* value threshold to consider significance.

**Figure 7:**
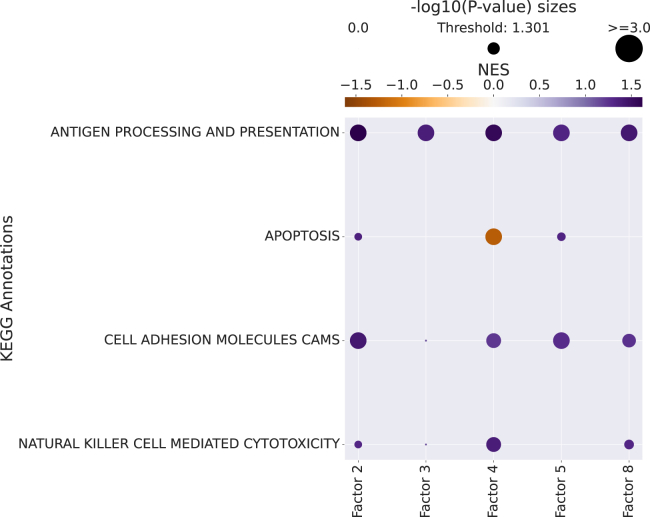
Assigning functions to factors from GSEA By using the loadings of ligand-receptor pairs per factor, they can be ranked within a factor (factor-specific analysis), and this information can be used to run an enrichment analysis such as GSEA to associate each of the programs with different functions or pathways. This dot plot shows the enriched KEGG pathways per factor. Dot size indicates the –log(*p* value), while the color indicates the normalized enrichment score (NES) from the GSEA.

Now that we have obtained the normalized enrichment scores (NESs) and corresponding *p* values from GSEA, we can plot those using the following function from cell2cell (Figure 7).

pathway_label = '{} Annotations'.format(pathwaydb)

fig_filename = output_folder + '/GSEA-Dotplot.pdf'

with sns.axes_style("darkgrid"):

 dotplot = c2c.plotting.pval_plot.generate_dot_plot(pval_df=pvals,

 score_df=scores,

 significance=0.05,

 xlabel='',

 ylabel=pathway_label,

 cbar_title='NES',

 cmap='PuOr',

 figsize=(5, 12),

 label_size=20,

 title_size=20,

 tick_size=12,

 filename=fig_filename

 )

#### Footprint enrichment analysis (timing: <1 min)

In footprint enrichment analysis, instead of considering the genes whose products (proteins) are directly involved in a process of interest, we consider the genes affected by it—i.e., those that change downstream as a consequence of the process.^45^ In this case, we will use the PROGENy resource to infer the pathways driving the identified context-dependent patterns of ligand-receptor pairs. PROGENy was built in a data-driven manner using perturbation data.^44^ Consequently, it assigns different weights to each gene in its pathway gene sets according to its importance. Thus, we need an enrichment method that can account for weights. To do so, we will use a multivariate linear regression implemented in decoupler-py.^43^

As we did in GSEA using Tensor-cell2cell, we first have to generate ligand-receptor gene sets while also assigning a weight to each ligand-receptor interaction. This is done by taking the mean between the ligand and receptor weights. For ligand and receptor complexes, we first take the mean weight for all subunits. We keep ligand-receptor weights only if all the proteins in the interaction are sign coherent and present for a given pathway. Load the PROGENy gene sets and then convert them to sets of weighted ligand-receptor pairs.

# We first load the PROGENy gene sets

net = dc.get_progeny(organism='human', top=5000)

# Then convert them to sets with weighted ligand-receptor pairs

lr_progeny = li.rs.generate_lr_geneset(lr_pairs, net, lr_sep="^")

Run footprint enrichment analysis using the “mlm” method from decoupler-py:

="^")

estimate, pvals = dc.run_mlm(lr_loadings.transpose(),

lr_progeny,

source="source",

target="interaction",

use_raw=False

Here, “estimate” and “pvals” correspond to the t values and *p* values assigned to each pathway.

Finally, we generate a heatmap for the 14 pathways in PROGENy across all factors (Figure S3A).

fig_filename = output_folder + '/PROGENy.pdf'

_ = sns.clustermap(estimate,

xticklabels=estimate.columns,

cmap='coolwarm',

z_score=4)

plt.savefig(fig_filename, dpi=300, bbox_inches='tight')

From the heatmap, we can also generate a bar plot for the PROGENy pathways for a specific factor (Figure S3B).

selected_factor = 'Factor 5'

fig_filename = output_folder + '/PROGENy-{}.pdf'.format(selected_factor.replace(' ', '-'))

dc.plot_barplot(estimate,

selected_factor,

vertical=True,

cmap='coolwarm',

save=fig_filename)

## Discussion

In this protocol, we illustrate how LIANA and Tensor-cell2cell can be used together to provide robust and flexible solutions to infer CCC programs across contexts. In addition to established methods for studying ligand-receptor interactions^19^^,^^32^ that LIANA also includes, approaches geared toward the systematic inference of CCC programs across diverse conditions are less common. A few of them, such as CellChat,^19^ summarize pathway-focused similarities across conditions based on pairwise comparisons, while MultiNicheNet^20^ depends on differential expression analysis and requires a hypothesis to be defined *a priori*. MultiNicheNet was recently proposed to systematically identify deregulated CCC interactions along with associated intracellular signaling. MultiNicheNet uses a flexible statistical framework and is capable of handling complex experimental designs. However, MultiNicheNet depends on differential expression analysis and hence requires a predefined hypothesis. As such, we see MultiNicheNet and Tensor-cell2cell as complementary, since the latter can identify patterns across all cell types and conditions in an untargeted manner. An analogous strategy to Tensor-cell2cell can be adopted by using factor analysis^11^ in LIANA to identify patterns directly from the CCC scores.^46^ Hence, Tensor-cell2cell and LIANA can help researchers to generate a specific hypothesis and identify cell types to later use MultiNicheNet as a downstream analysis to additionally infer intracellular signaling triggered by key ligands.

Since our pipeline is intended as a generalizable approach for use with many different resources and methods, we additionally assessed the robustness of our results across different inputs. Specifically, we showed how communication scores may be different for individual samples across methods (see Tutorial 02 in the online tutorials), whereas those differences may be mitigated by using the consensus score or when running Tensor-cell2cell across multiple samples (see Python Supplementary Tutorials S3A-2 and S3B in the online tutorials). Moreover, we provide an in-depth assessment of Tensor-cell2cell’s sensitivity to missing values and batch effects (STAR Methods). Additional benchmarks can be found in the original Tensor-cell2cell^12^ and recent LIANA+^46^ articles, where we have shown that Tensor-cell2cell consistently captures CCC events deregulated across diverse contexts and conditions. Finally, we demonstrate the broad applicability of our protocol by also providing an example of defining contexts to analyze CCC using spatial transcriptomics (see STAR Methods and Python Supplementary Tutorial S4 in the online tutorials). Although the example using spatial transcriptomics presented in our extended tutorials is a simplified application of the concept, it could be extended to compare multiple samples if users are able to align tissues from different donors. Similarly, our protocol can also aid users in applications beyond single-cell transcriptomics data, including extracting metabolite-mediated CCC programs^27^ or similar extensions to multiomics data.^46^

### Limitations of the study

Similar to any other approach to infer CCC from transcriptomics data, our protocol also inherits assumptions leading to certain limitations. These include the assumption that gene co-expression is indicative of active signaling events, which are largely mediated by proteins and their interactions, while also disregarding multiple biological processes, such as protein translation, post-translational modifications, secretion, diffusion, and trigger of intracellular events, that precede and follow the interaction itself.^2^^,^^5^ Moreover, the aggregation of single cells into cell groups is essential when inferring potential CCC events, which could occlude some signals in heterogeneous tissues,^2^^,^^3^ thereby biasing the insights that can be obtained. Furthermore, the input of Tensor-cell2cell is a 4D tensor, so it requires that all elements be measured across all features and samples (i.e., cell types and genes expressing ligands and receptors). Consequently, one should consider how to handle missing values across samples that do not capture the same cell types and/or expressed genes. Deciding whether those reflect biologically meaningful zeros or a technical artifact may lead to variations in the resulting CCC programs. We provide an extended explanation of the related parameter choices that may help users decide how to handle this challenge (STAR Methods).

## STAR★Methods

### Key resources table

**Table:** 

REAGENT or RESOURCE	SOURCE	IDENTIFIER
**Deposited data**		

COVID BALF single-cell RNA-seq dataset	Liao et al.^30^	GEO: GSE145926; Zenodo Data: https://doi.org/10.5281/zenodo.7706962
PBMC single-cell RNA-seq dataset	Kang et al.^47^	GEO: GSE96583; Zenodo Data: https://doi.org/10.5281/zenodo.10069528
Myocardial Infarction spatial transcriptomics dataset	Kuppe et al., 2022^22^	Zenodo Data: https://doi.org/10.5281/zenodo.6578047

**Software and algorithms**		

Protocol source code	This paper	https://doi.org/10.5281/zenodo.10700956
Code for benchmarking batch effects and missing values	This paper	https://doi.org/10.5281/zenodo.10713331

### Resource availability

#### Lead contact

Further information and requests for resources should be directed to and will be fulfilled by the lead contact, Nathan E. Lewis (nlewisres@ucsd.edu).

#### Materials availability

This study did not generate new unique reagents.

#### Data and code availability

• This paper analyzes existing, publicly available data. These accession numbers for the datasets are listed in the key resources table. In particular, the BALF single-cell RNA-seq dataset is available at https://zenodo.org/record/7706962, the PBMC single-cell RNA-seq dataset is available at https://zenodo.org/records/10069528, and the Myocardial Infarction spatial transcriptomics dataset is available at https://zenodo.org/record/6578047.
• All original code has been deposited at Zenodo and is publicly available as of the date of publication. DOIs are listed in the key resources table. Additionally, source code is available at https://github.com/saezlab/ccc_protocols and can be viewed at https://ccc-protocols.readthedocs.io/.
• Any additional information required to reanalyze the data reported in this paper is available from the lead contact upon request.

### Method details

#### Computational Infrastructure

All code was ran on a computer with the following specifications.

• CPU: AMD Ryzen Threadripper 3960x (24 cores)
• Memory: 128GB DDR4
• GPU: NVIDIA RTX A6000 48GB

However, the minimal requirements for running this protocol are.

• CPU: 64-bit Intel or AMD processor (4 cores)
• Memory: 16GB DDR3
• GPU: NVIDIA GTX 1050 Ti (Optional)
• Storage: At least 10GB available

#### Timing

Expected timing for this protocol using the dataset in the key resources table:

Step 1. Installation of Anaconda/Miniconda and Python packages: 5–30 min.

Step 2. Initial setups: ∼1 min.

Step 3. Data preprocessing: 5–7 min.

Step 4. Inferring cell-cell communication with LIANA: ∼5 min.

Step 5. Comparing cell-cell communication across multiple samples with Tensor-cell2cell: Running selection of number of factors via elbow analysis and the tensor decomposition takes 5 min with the ‘regular’ pipeline, while the ‘robust’ pipeline takes 40 min.

Step 6. Functional Enrichment Analysis of KEGG and PROGENy pathways respectively using GSEA and linear regression take 1 min each.

#### Protocol details

To run our protocol presented in this manuscript and the tutorials available online (https://ccc-protocols.readthedocs.io/), software specifications are summarized in the Software Requirements Table. To facilitate the setup of a virtual environment containing all required packages with their corresponding versions, we provide an executable `setup_env.sh` script together with instructions on a Github repository we prepared for this protocol: https://github.com/saezlab/ccc_protocols/tree/main/env_setup.

Software Requirements Table

**Table:** 

Package Name	Package Version	Language	Install With
jupyter			conda
ipywidgets			conda
pip	≥22	Python	conda
scanpy	≥1.9	Python	conda
∗cuda-toolkit			conda
∗pytorch-cuda	11.8		conda
∗torchvision			conda
∗torchaudio			conda
pytorch, ∗cuda enabled			conda
scvi-tools	≥0.18	Python	conda
scikit-misc	0.1.4	Python	conda
cell2cell	0.7.3	Python	pip
liana	1.0.3	Python	pip
decoupler	1.5.0	Python	pip
omnipath	1.0.7	Python	pip
plotnine	≥0.12.4	Python	pip
seaborn	0.11.2	Python	pip
statannotations	0.5.0	Python	pip
matplotlib	3.7.3	Python	pip
singlecellexperiment		R	conda
remotes	≥2	R	conda
devtools	≥2	R	conda
seuratobject		R	conda
biocmanager	≥1.30	R	conda
seurat	≥4	R	conda
hd5r		R	conda
furrr		R	conda
textshape		R	conda
forcats		R	conda
rstatix		R	conda
ggpubr		R	conda
scater		R	conda
zellkonverter		R	conda
liana	0.1.13	R	remotes
seurat-disk	0.0.0.9020	R	remotes
decoupleR	2.3.3	R	biocmanager

∗: For GPU enabled use only.

Python packages should always be installed. R language packages only need to be installed if planning to run the notebooks in R.

Advice to deal with potential issues running this protocol, either in its original or personalized forms, is summarized in the Troubleshooting Table.

Troubleshooting Table.

**Table:** 

Step	Problem	Possible reason	Solution
3 & 4	Error: Expression matrix contains non-finite values (nan or inf) Warning: Make sure that normalized counts are passed	Mishandling counts processing	Ensure that the matrix containing normalized counts is passed. Replace nan and inf values by zeros.
4.1	Negative values in LIANA outputs	Using preprocessed data with negative expression values.	Avoid using preprocessing methods that generate negative values (e.g., centering the data to the mean values, using batch-corrected expression values, etc.).
4.2	Not enough ligand-receptor pairs in the data for the analysis	Mismatched symbol IDs	LIANA by default uses a resource with gene symbol IDs. When working with e.g., Ensembl IDs users need to provide an external resource; see https://ccc-protocols.readthedocs.io/en/latest/notebooks/ccc_python/02-Infer-Communication-Scores.html
5.1	CCC scores representing opposed importance	When using ‘magnitude_rank’ scores from LIANA, lower values are more important. However, Tensor-cell2cell prioritizes high values as the important ones.	Build the 4D tensor using an `inverse_fun` to make lower values to be the most important scores.
5.2	Rank selection through the elbow analysis is not behaving properly	High sparsity or number of missing values in the tensor	Re-run LIANA with less stringent parameters (e.g., smaller expr_pror). Re-build the tensor with more strict how parameters (e.g., using how = ‘inner’ or increasing outer_fraction).
5.3	Visualization of loadings are not properly displayed in heatmaps	Too many or few elements in the dimension to visualize	To visualize all elements, use the parameter `loading_threshold = 0′ to create the heatmaps. If you have too many elements, you can prioritize those with high loadings, so a threshold can be set. E.g., `loading_threshold = 0.1′

#### Benchmarking batch effects and missing values

To help users make informed decisions regarding choices in their computational pipeline, we benchmarked two key factors that can influence Tensor-cell2cell′s outputs: batch effects and missing data (which result in missing tensor indices) across samples. For comprehensive details on the motivation, methods, and results of this benchmarking, please see the online description.^48^

Here we describe our pipeline for both the Missing Indices and Batch Effects benchmarking simulations. All associated code can be found in the following repository: https://github.com/hmbaghdassarian/tc2c_benchmark. For downstream analyses, unless otherwise specified, all linear regressions were performed using a generalized linear model (GLM) with an identity link function; multivariate regressions with >1 independent variable were combined additively and do not include interaction terms. Additionally, all *p*-values were multiple-test-corrected using the Benjamini-Hochberg (BH) method to control for false discovery rates (FDRs).

We simulated single-cell RNA-sequencing expression data using Splatter,^49^ adapting a previously described computational approach.^50^ We generated a single-cell expression matrix containing 2000 genes and 5000 cells evenly distributed across 6 cell types and 5 samples. Each sample represents a context.

Next, for each sample, we applied quality control filters to the cells and genes as implemented previously.^50^ Briefly, low-quality cells were identified and filtered using the scuttle package based on standard metrics (mitochondrial fraction, library size, and number of genes detected); genes detected in fewer than 1% of cells are discarded. Next, counts were normalized using scran pooling^51^ and a log_+1_ transformation. For batch-effect benchmarking, batch correction was further implemented; Scanorama^52^ was run on the log-normalized counts matrix and scVI^53^ was run on the raw counts matrix.

From the expression counts matrices, a random subset of 200 genes were chosen to simulate a ligand-receptor interaction network as previously described.^12^ Briefly, we use StabEco’s^54^ BiGraph function, with the power law exponent value set to 2 and the average degree value set to 3, to generate a scale-free, directed, bipartite network of the 200 genes. Half the genes were assigned to be ligands and the other half to be receptors. Not all genes were part of the connected network (70/200), and these were excluded from downstream analyses. This interaction network was used as custom ligand-receptor resource input to LIANA’s cell-cell communication scoring.

Then, 4D-Communication tensors were built from the output of LIANA as described in our protocol. To generate missing indices in the 4D-Communication Tensor, we iteratively omitted a random subset of genes or cell types from the expression data. Specifically, we iterated through combinations of the following two variables: the fraction of cell types to remove in a given sample (16, 13, 12, and 23), the fraction of genes (within the 130 in the simulated LR interaction network) to remove in a given sample (110, 310, 12), and the fraction of samples to apply these omissions to (15, 25, 23). We compared this to a gold-standard tensor with no missing indices.

We compared decomposition outputs using CorrIndex^55^ as previously described.^12^ Briefly, the CorrIndex represents a dissimilarity between decomposition outputs and lies between 0 and 1; we convert this to a similarity metric by using (1-CorrIndex).

For batch-correction, iterating across increasing levels of batch severity, we generated four counts matrices.

(1) Gold-standard: a processed counts matrix with no batch effects
(2) Log-normalized: a processed counts matrix with batch effects present
(3) Scanorama batch-corrected: a processed counts matrix with batch effects corrected for using Scanorama
(4) scVI batch-corrected: a processed counts matrix with batch effects corrected for using scVI

We ran the combined LIANA and Tensor-cell2cell pipeline on each of these counts matrices. Finally, we assessed the similarity between each of the decomposition outputs as follows.

• Log-normalized similarity: Similarity between Tensor-cell2cell′s decomposition output from the log-normalized counts matrix (2) and that of the gold-standard input (1)
• Scanorama similarity: Similarity between Tensor-cell2cell′s decomposition output from the Scanorama batch-corrected counts matrix (3) and that of the gold-standard input (1)
• scVI similarity: Similarity between Tensor-cell2cell′s decomposition output from the scVI batch-corrected counts matrix (4) and that of the gold-standard as input (1)

Additionally, for batch correction benchmarking, each counts matrix was quantified for its level of batch severity using two previously applied metrics^50^^,^^56^: (1) kBET,^57^ is an inverse measure of “mixability”, or the extent to which batch effects are removed, and (2) normalized mutual information (NMI) between cell type identity and cluster identity - a measure of “clusterability”, or the extent to which biological variation is conserved. For the clusterability metric, we subtracted the NMI from 1 to quantity batch severity. In this manner, both mixability and clusterability ranged between 0 and 1, with increasing values indicating increasing batch severity. Clusterability was assessed using both k-means clustering^58^ and Louvain clustering.^59^

Batch severity does not affect the results of our pipeline. We saw that the gold-standard matrix performed as expected, showing clear Louvain clusterability and little-to-no mixability. The log-normalized matrix also performed as expected across all batch severity metrics. While the batch-corrected counts matrices increased along with the Splatter parameters on occasion, the increases were overall less severe than that of the log-normalized matrix (Figure S4). The gold-standard counts matrices demonstrate comparably low batch severity across all iterations (Figure S5A). We also saw that across all batch severity metrics, similarity does not decrease beyond 0.963, indicating that Tensor-cell2cell is robust to batch effects (Figure S5B). Furthermore, we evaluated whether the fraction of negative counts is a confounder of batch severity (Figures S5C–S5E). The fraction of negative counts does not substantially affect the Scanorama similarity as indicated by the small regression coefficient estimate and insignificant *p*-value (Figure S5F). This tells us that using batch correction methods that introduce negative values and simply replacing those with 0 prior to running communication scoring can be appropriate for recovering biological signals from Tensor-cell2cell.

If batch correction improves decomposition, we would expect batch-corrected similarity (Scanorama and scVI) to a) score higher than log-normalized similarity across batch similarity metrics and b) decrease at a lower rate with increasing batch severity than log-normalized similarity. Across batch severity metrics, we see that this tends not to be the case, though all similarity types maintain a high similarity score across batch severity levels (Figure S6) Overall, while batch effect correction may not be necessary to recover biological signals using Tensor-cell2cell, if the user feels it is important, they can be comfortable in implementing the batch correction method of choice.

Regarding missing values, we found that there was a significant decrease in the similarity of Tensor-cell2cell′s output with that of the gold-standard as the fraction of missing indices increased when filling both with NaN (masked) or zero (not masked). However, those that were not masked had a substantially larger decrease in similarity than those that were (Figure S7A). When considering the two filling methods in combination with the missing fraction, we see that similarity is lower by 0.094 on average when filling with zero (Figure S7B). Altogether, our pipeline is robust enough to impute missing values and sensitive enough to handle true biological zeros.

### Quantification and statistical analysis

#### Notations for the scoring functions in LIANA

k is the k-th ligand-receptor interaction

L - expression of ligand L

R - expression of receptor R

C - cell cluster

i - cell group i

j - cell group j

M - the library-size normalized and log1p-transformed gene expression matrix

X - normalized gene expression vector

We denote the two interaction proteins, via their genes L & R, yet we use this for convenience as these can also denote the interaction of any other event category, such as those between membrane-bound or extracellular matrix proteins. Furthermore, in the case of heteromeric complexes L & R denote the summarized expression of the complex.

CellPhoneDBv2^32^ function.

Magnitude**:** 1) ${LRmean}_{k,i,j}=\frac{L_{C_{i}}+R_{C_{j}}}{2}$

Specificity**:** A permutation approach also adapted by other methods, see 4)

Geometric Mean function.

Magnitude: 2) ${LRgeometric.mean}_{k,i,j}=\sqrt{L_{C_{i}\cdot }R_{C_{j}}}$

Specificity**:** An adaptation of CellPhoneDB’s permutation approach; see 4)

CellChat’s^19^ LR probabilities∗ function.

Magnitude: 3) $LR{prob}_{k,i,j}=\frac{TriMean\left(L_{c_{i}}\right)\cdot TriMean\left(R_{c_{j}}\right)}{Kh+TriMean\left(L_{c_{i}}\right)\cdot TriMean\left(R_{c_{j}}\right)}$

where *Kh* = 0.5 by default and `TriMean` represents Tuckey’s Trimean function:

$$TriMean\;\left(X\right)=\frac{Q_{0.25}\left(X\right)+2\cdot Q_{0.5}\left(X\right)+Q_{0.75}\left(X\right)}{4}$$

Specificity: An adaptation of CellPhoneDB’s permutation approach; see 4)

**∗Note:** The original CellChat implementation uses information of mediator proteins (e.g. activators and inhibitors) and signaling pathways, which is specific to the CellChat resource. Since LIANA allows combining any resource with different scoring methods, LIANA does not utilize this information, and hence the implementation of CellChat's scoring function in LIANA was simplified to be resource-agnostic.

Equation 4 $${p-value}_{k,i,j}=\frac{1}{P}\sum_{p=1}^{P}\left[{fun}_{permuted}\left(L_{C_{i}}^{*},R_{C_{j}}^{*}\right)\geq {fun}_{observed}\left(L_{C_{i}}^{*},R_{C_{j}}^{*}\right)\right]$$

where P is the number of permutations, and L∗ and R∗ are ligand and receptor expressions summarized according to the aggregation function per cluster used by each method, i.e., by default the arithmetic mean for CellPhoneDB and Geometric Mean, and TriMean for CellChat.

SingleCellSignalR^35^ function.

Magnitude**:** 5) ${LRscore}_{k,i,j}=\frac{\sqrt{L_{C_{i}}R_{C_{j}}}}{\sqrt{L_{C_{i}}R_{C_{j}}}+\mu }$

where μ is the mean of the expression matrix M

NATMI^34^ function.

Magnitude: 6) ${LRproduct}_{k,i,j}=L_{C_{i}}R_{C_{j}}$

Specificity: 7) ${Specificity\;Weight}_{k,i,j}=\frac{L_{C_{i}}}{\sum^{n}L_{C_{i}}}\cdot \frac{R_{C_{i}}}{\sum^{n}R_{C_{j}}}$

Connectome^33^ function.

Magnitude: 6) ${LRproduct}_{k,i,j}=L_{C_{i}}R_{C_{j}}$

Specificity: 8) $LRz.{mean}_{k,i,j}=\frac{z_{L_{C_{i}}}+z_{R_{C_{j}}}}{2}$

where z is the *Z* score of the expression matrix M

Log2FC function.

Specificity: 9) ${LRlog2FC}_{K,I,J}=\frac{{\mathrm{Log}\;2FC}_{C_{i,L}}+{\mathrm{Log}\;2FC}_{C_{j,R}}}{2}$

where log2FC for each gene is calculated as:

Equation 10 $$\log\;2\;FC=\log_{2}\left(mean\;\left(X_{i}\right)\right)-\log_{2}\left(mean\left(X_{{not}_{i}}\right)\right)$$

Rank Aggregate function.

When generating a consensus from the different methods in LIANA, a rank aggregate^36^ is calculated for the *magnitude* and *specificity* scores from the methods separately. First, a normalized rank matrix[0,1] is generated separately for magnitude and specificity as:

Equation 11 $$r_{i,j}=\frac{\mathrm{ran}k_{i,j}}{\max\left(\mathrm{ran}k_{i,j}\right)}\left(1\leq i\leq m,1\leq j\leq n\right)$$

where m is the number of ranked score vectors, n is the length of each score vector (number of interactions), $\mathrm{ran}k_{i,j}$ is the rank of the j-th element (interaction) in the i-th score rank vector, and $\max\left(ra{nk}_{i}\right)$ is the maximum rank in the i-th rank vector.

For each normalized rank vector r, we then ask how probable it is to obtain $r_{\left(k\right)}^{null}<=r_{\left(k\right)}$, where $r_{\left(k\right)}^{null}$ is a rank vector generated under the null hypothesis. The RobustRankAggregate (https://github.com/cran/RobustRankAggreg) method expresses the probability $r_{\left(k\right)}^{null}<=r_{\left(k\right)}$ as $\beta_{k,n}\left(r\right)$ through a beta distribution. This entails that we obtain probabilities for each score vector r as:

Equation 12 p(r)=βk,n1,⋯,nmin(r)∗n

where we take the minimum probability ρ for each interaction across the score vectors, and we apply a Bonferroni correction to the *p*-values by multiplying them by n to account for multiple testing.

For all the methods above, LIANA considers interactions as occurring only if the ligand and receptor, and all of their subunits, are expressed in a certain proportion of the cells (0.1 by default) in both clusters involved in the interaction. This can be formulated as an indicator function as follows:

$$I\left\{L_{C_{j}}^{expr.prop}\geq 0.1\;and\;R_{C_{j}}^{expr.prop}\geq 0.1\right\}$$

## Supplemental information

Document S1. Figures S1–S7

Document S2. Article plus supplemental information
