# Supplementary material for: Combining LIANA and Tensor-cell2cell to decipher cell-cell communication across multiple samples
Source: Cell Rep Methods. 2024 Apr 16;4(4):100758. doi: 10.1016/j.crmeth.2024.100758 (PMC11046036; doi:10.1016/j.crmeth.2024.100758)
Supplement: Document S1. Figures S1–S7 [file mmc1.pdf]

Cell Reports Methods, Volume *4*

Supplemental information

Combining LIANA and Tensor-cell2cell to decipher cell-cell communication across multiple samples

Hratch M. Baghdassarian, Daniel Dimitrov, Erick Armingol, Julio Saez-Rodriguez, and Nathan E. Lewis

Supplemental information

Combining LIANA and Tensor-cell2cell to decipher cell-cell communication across multiple samples

Hratch M. Baghdassarian, Daniel Dimitrov, Erick Armingol, Julio Saez-Rodriguez, Nathan E. Lewis

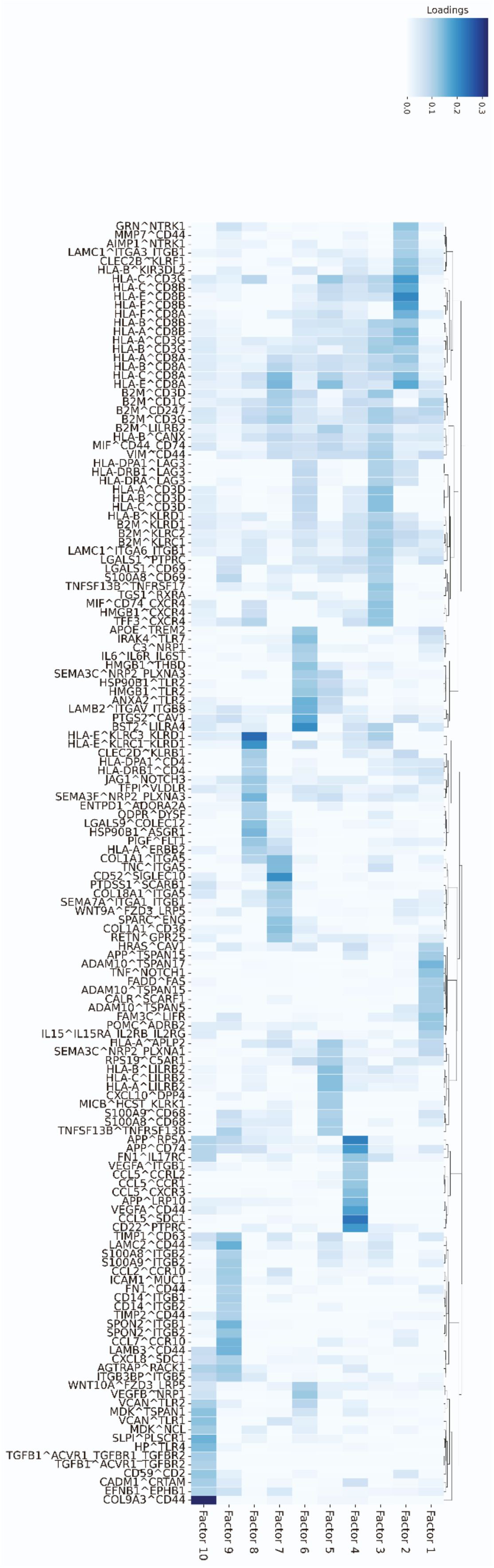

Figure S1. Inspecting important ligand-receptor pairs per factor, related to Figure 5. Clustermap of key ligand-receptor pairs whose loadings are above a threshold can be clustered depending on their importance across all cell-cell communication programs. Columns are factors while rows are distinct ligand-receptor pairs. Colors represent their respective loadings.

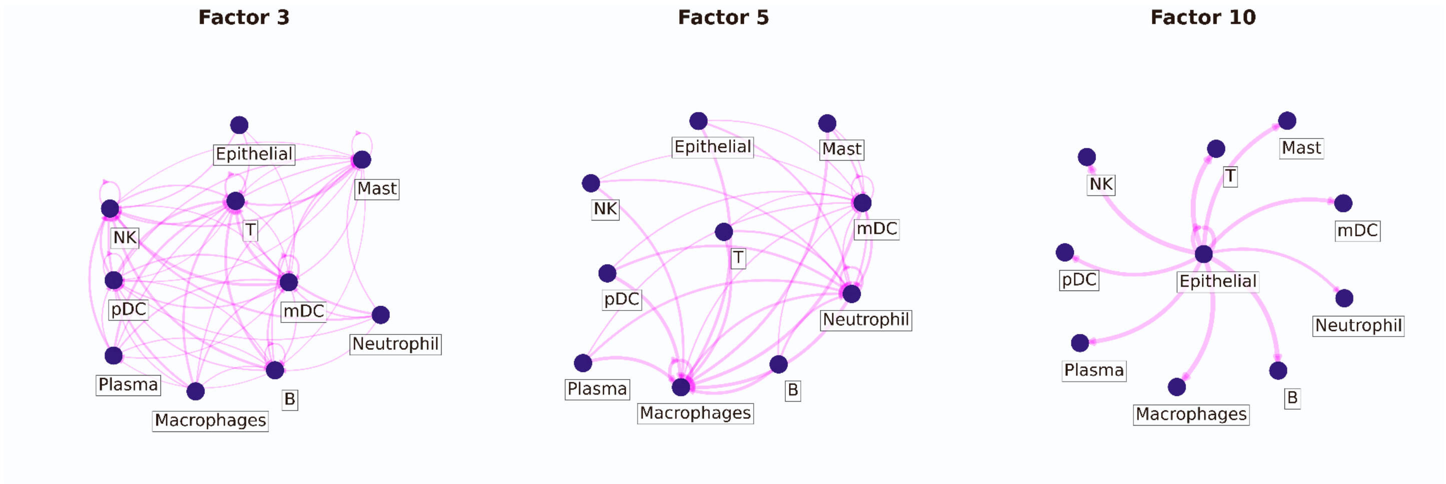

Figure S2. Factor-specific networks of overall cell-cell interactions, related to Figure 5. Cell type loadings can be jointly used within a factor to have an overall representation of the cell-cell communication (i.e., a factor-specific network of communication). Adjacency matrices are computed per factor by computing the outer product between the sender and the receiver loadings within each factor, which later are used to visualize the networks. A threshold can be set to show important interactions that each factor captures.

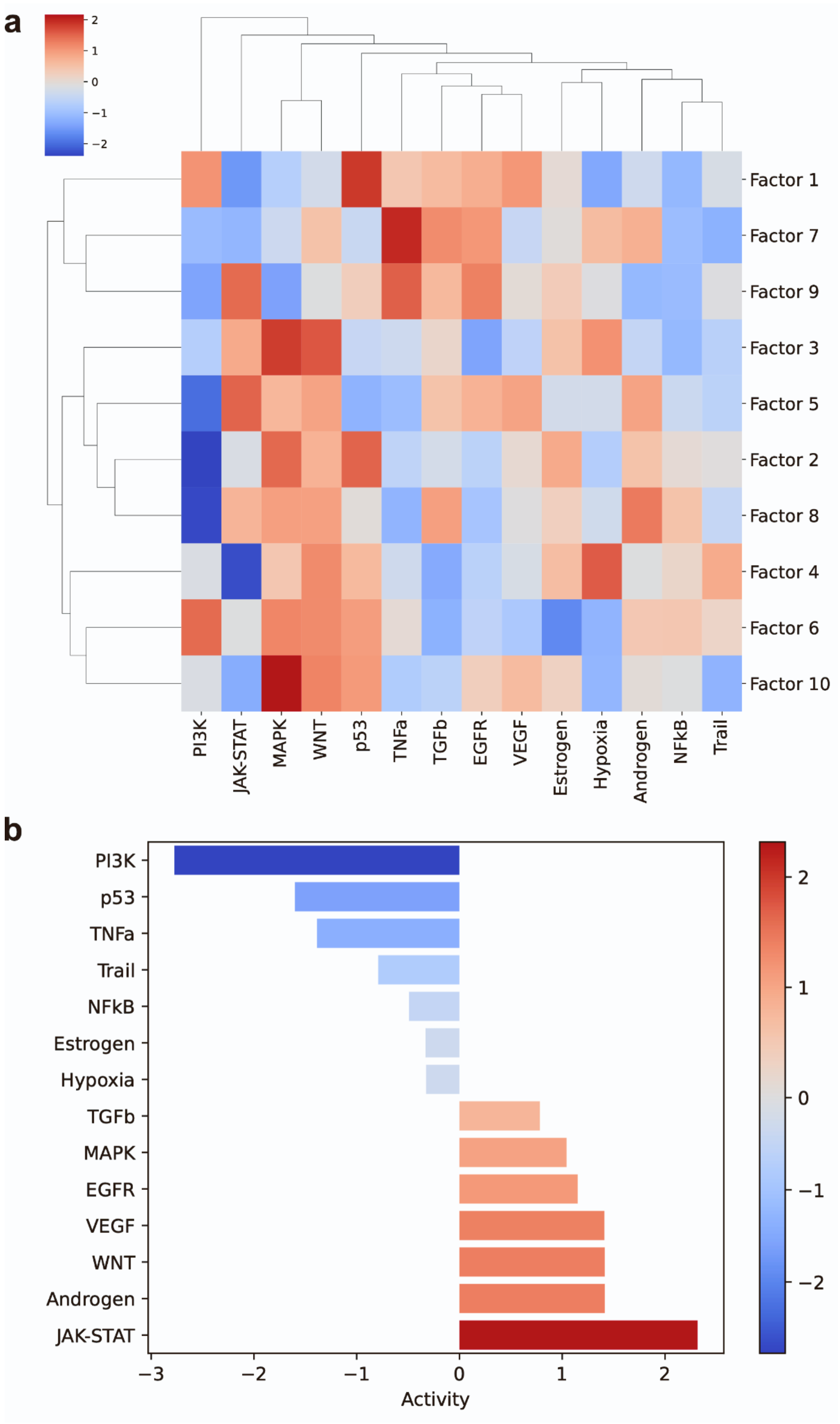

Figure S3. Assigning functions to factors with PROGENy, related to Figure 5. By using the loadings of ligand-receptor pairs per factor, they can be ranked within a factor (factor-specific analysis), and this information can be used to infer pathway activity from PROGENy to associate each of the programs with different signaling pathways (**a**). Activity of different pathways can be further inspected and easily compared with a bar plot (**b**), as shown here for factor 5.

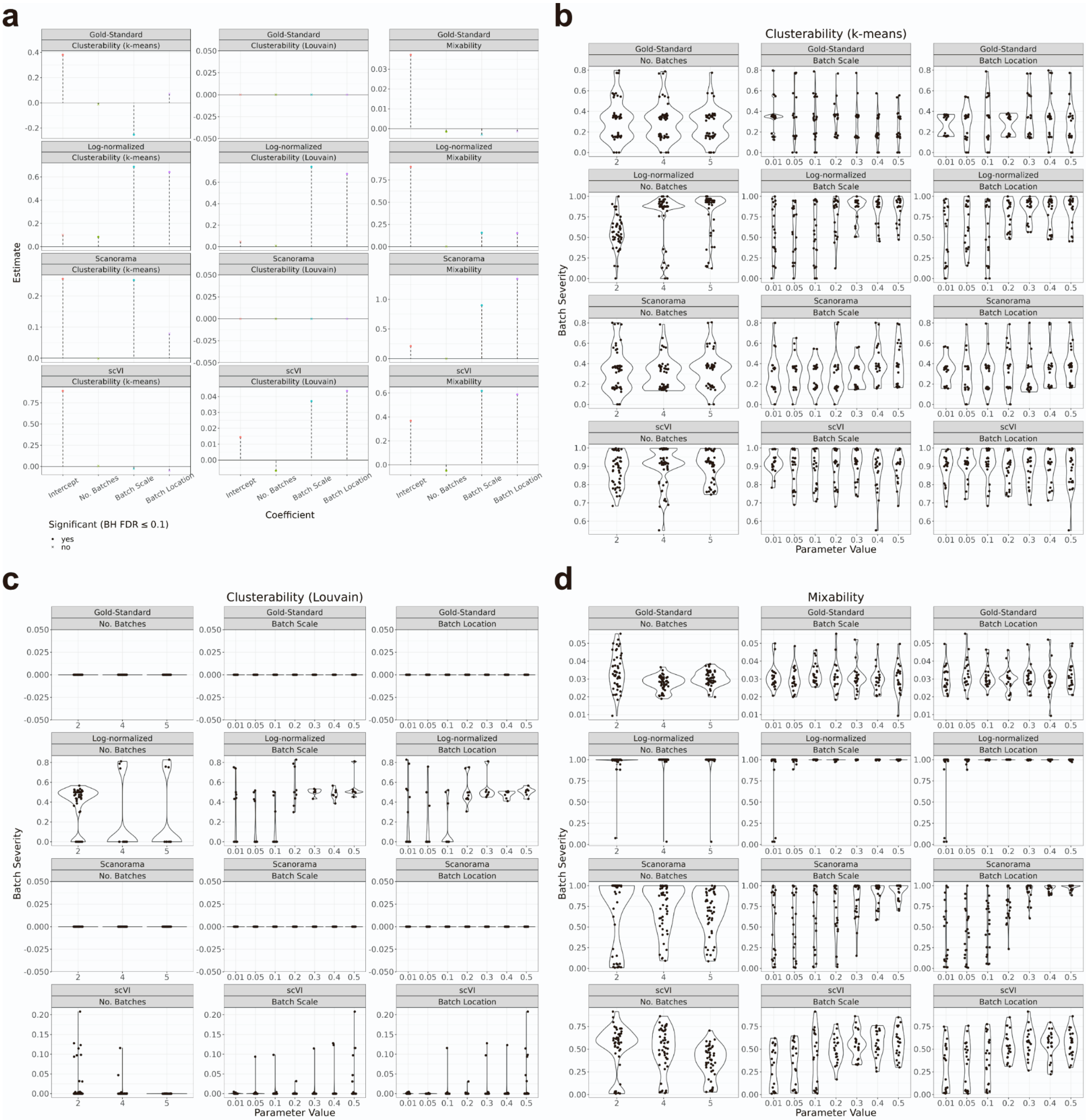

Figure S4. Effect of Splatter parameters, related to Figure 2. (**a**) Coefficient estimates of linear regressions estimating batch severity (dependent variable) from the Splatter parameters used to introduce batch effects (independent variables). Panels represent distinct processed counts matrix inputs (rows) and batch severity metrics (columns). Coefficient estimates are on the y-axis, coefficient labels are on the x-axis, and the horizontal solid black line represents 0 (no effect). Significant estimates (Wald test, Benjamini-Hochberg FDR correction, q-value ≤ 0.1) are marked by a circle and insignificant estimates are marked by a cross. (**b**) Violin plots of batch severity (y-axis) as measured by k-means clusterability across iterations. Panels represent distinct processed counts matrix inputs (rows) and Splatter parameters (columns). (**c**) Violin plots of batch severity (y-axis) as measured by Louvain clusterability across iterations. Panels represent distinct processed counts matrix inputs (rows) and Splatter parameters (columns). (**d**) Violin plots of batch severity (y-axis) as measured by mixability across iterations. Panels represent distinct processed counts matrix inputs (rows) and Splatter parameters (columns).

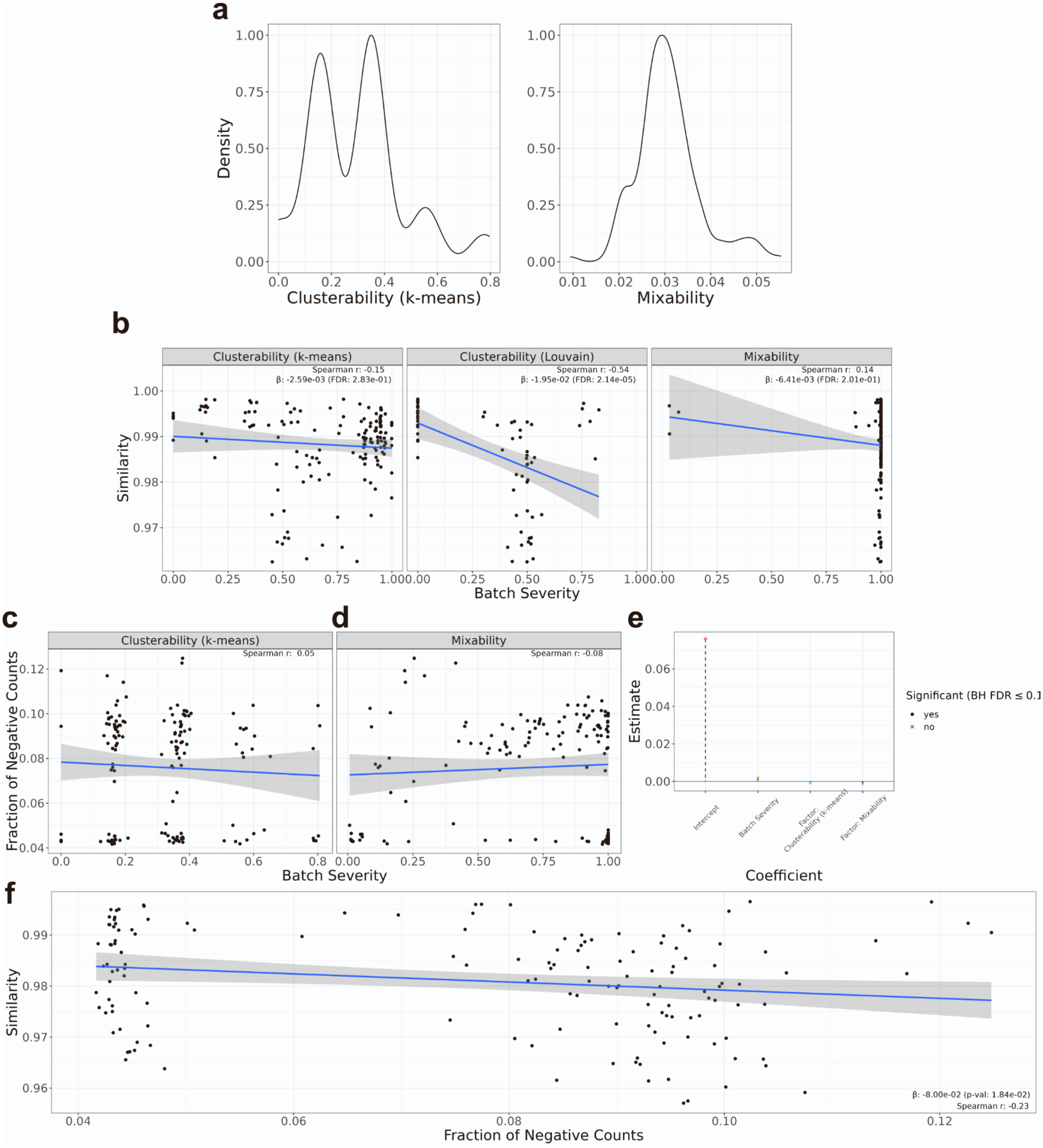

Figure S5. Impact of batch effects under different batch severities, related to Figure 2. (**a**) Distributions of the batch-severity introduced to the gold-standard counts matrix. Scaled kernel density estimates (y-axis) are shown for batch severity (x-axis) measured in two ways: by k-means clusterability (left panel) and mixability (right panel). (**b**) Robustness of Tensor-cell2cell to batch effects. Scatterplots of log-normalized similarity (y-axis) across batch severity (x-axis) assessed by three metrics (panels). Blue lines show the regression fit with gray shaded regions showing the 95% confidence interval for this fit. Spearman correlations and regression coefficient estimates (as well as their Wald test FDRs) are annotated in the upper right corner of each panel. (**c-f**) Introduction of negative counts by batch correction has negligible impact on the Tensor-cell2cell pipeline. Scatterplots and regression fits of the fraction of negative values (y-axis) introduced by Scanorama to the batch-corrected counts matrix across batch severity (x-axis) assessed by **(c)** k-means clusterability and **(d)** mixability. **(e)** Coefficient estimates of a linear regression estimating the fraction of

negative values (dependent variable) from batch severity while controlling for the batch severity metric (independent variables). Since Louvain clusterability is constantly 0 for all fractions of negative values, it is set as the reference. Coefficient estimates are on the y-axis, coefficient labels are on the x-axis, and the horizontal solid black line represents 0 (no effect). Significant estimates (Wald test, Benjamini-Hochberg FDR correction, q-value ≤ 0.1) are marked by a circle and insignificant estimates are marked by a cross. **(f)** Scatterplots and regression fit the Scanorama similarity (y-axis) across the fraction of negative values (x-axis). The regression coefficient estimate (as well as its Wald test p-value) are annotated in the bottom right corner. For all scatter plots and regression fits, Spearman correlations are annotated and blue lines show the regression fit with gray shaded regions showing the 95% confidence interval for this fit.

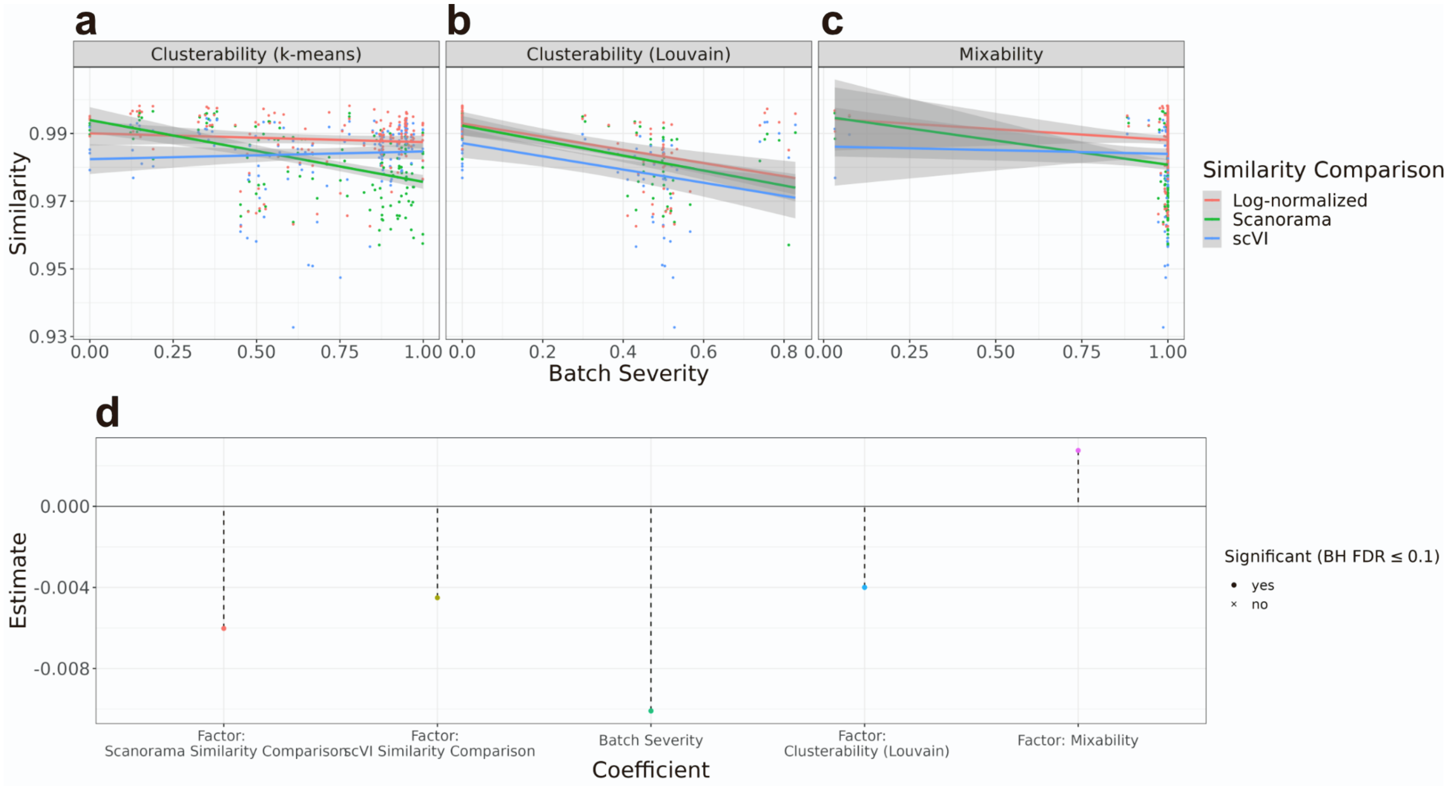

Figure S6. Batch effect correction has negligible impact on the Tensor-cell2cell pipeline, related to Figure 2. Scatterplots and regression fits of the fraction of decomposition similarity (y-axis) between log-normalized (red), Scanorama (green), and scVI (blue) and the gold-standard across batch severity (x-axis) assessed by **(a)** k-means clusterability, **(b)** Louvain clusterability, and **(c)** mixability. Solid lines show the regression fit with gray shaded regions showing the 95% confidence interval for this fit. **(d)** Coefficient estimates of a linear regression estimating the decomposition similarity (dependent variable) according to the similarity comparison type (independent variable) (i.e., log, Scanorama, or scVI) while controlling for batch severity and the batch severity metrics (independent variables). The log similarity comparison type is set as the reference to identify how batch correction compares. Coefficient estimates are on the y-axis, coefficient labels are on the x-axis, and the horizontal solid black line represents 0 (no effect). Significant estimates (Wald test, Benjamini-Hochberg FDR correction, q-value ≤ 0.1) are marked by a circle and insignificant estimates are marked by a cross. The regression intercept is 0.995 and significant (q << 1e-10) (data not shown).

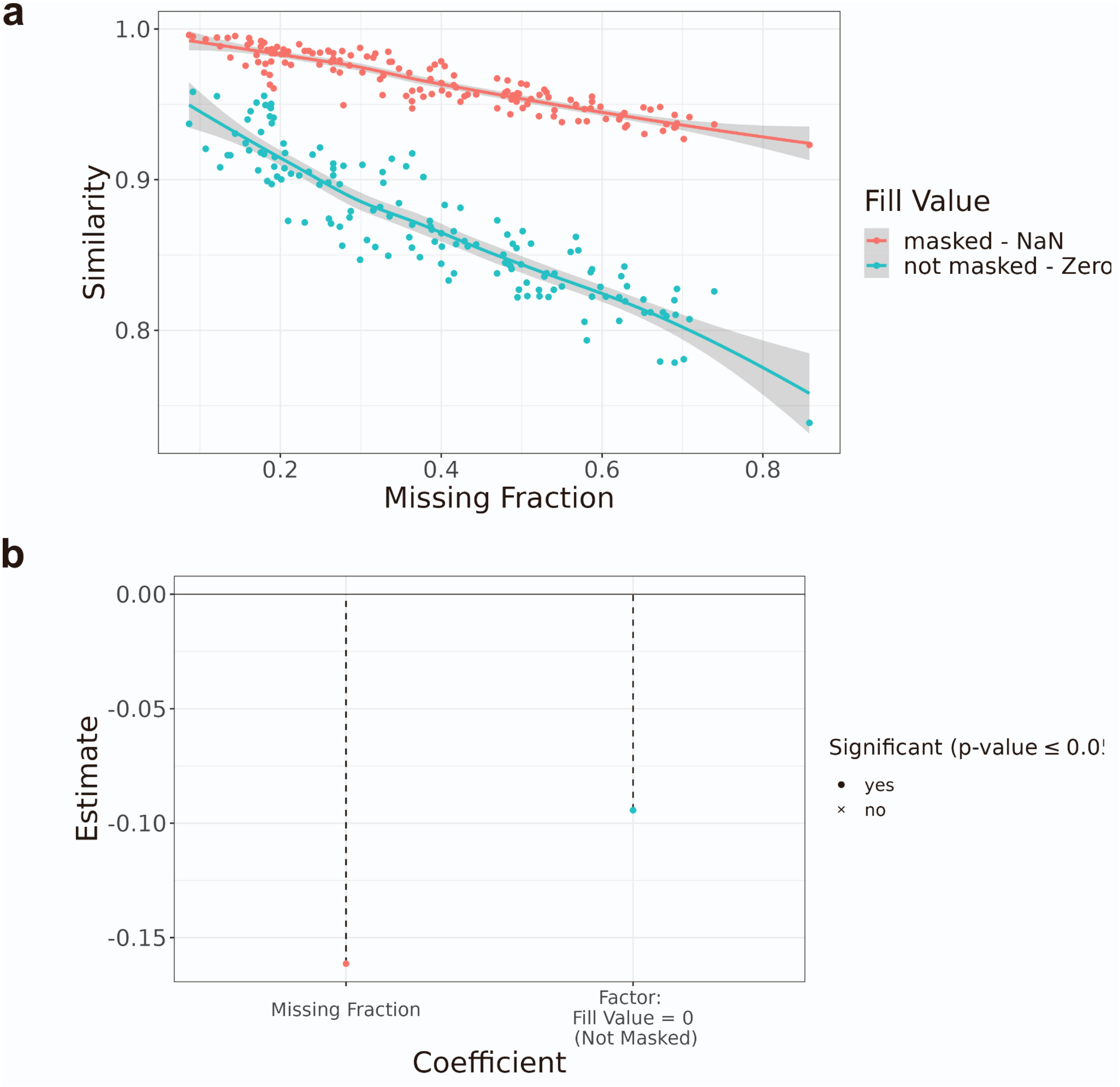

Figure S7. Effect of missing indices on Tensor-cell2cell's results, related to Figure 2. Tensor-cell2cell's output decreases as the fraction of missing elements in the tensor increases. (**a**) Scatterplots and regression fits of the decomposition similarity (y-axis) between the gold-standard tensor and that with missing indices filled with NaN and subsequently masked (red) or filled with 0 and not masked (green) across the fraction of missing elements in the tensor (x-axis). Solid lines show the regression fit with gray shaded regions showing the 95% confidence interval for this fit. (**b**) Coefficient estimates of a linear regression estimating the decomposition similarity (dependent variable) according to the fraction of missing elements in the tensor and the value those missing elements were filled with (independent variables). The NaN fill type (masking) is set as the reference since it is expected to affect similarity less. Coefficient estimates are on the y-axis, coefficient labels are on the x-axis, and the horizontal solid black line represents 0 (no effect). Significant estimates (Wald test, $p \leq 0.05$) are marked by a circle and insignificant estimates are marked by a cross. The regression intercept is 1.03 and significant ($p << 1e-10$) (data not shown).
